# The simulation-cum-ROC approach: A new approach to generate tailored cutoffs for fit indices through simulation and ROC analysis

**DOI:** 10.3758/s13428-025-02638-x

**Published:** 2025-04-01

**Authors:** Katharina Groskurth, Nivedita Bhaktha, Clemens M. Lechner

**Affiliations:** 1 Graduate School of Economic and Social Sciences, University of Mannheim, Mannheim, Germany; 2 GESIS – Leibniz Institute for the Social Sciences, Mannheim, Germany; 3 Indian Institute of Technology Kanpur, Kanpur, Uttar Pradesh India

**Keywords:** Fit indices, Cutoff, Confirmatory factor analysis, Structural equation modeling, ROC

## Abstract

To evaluate model fit in structural equation modeling, researchers commonly compare fit indices against fixed cutoff values (e.g., CFI ≥ .950). However, methodologists have cautioned against overgeneralizing cutoffs, highlighting that cutoffs permit valid judgments of model fit only in empirical settings similar to the simulation scenarios from which these cutoffs originate. This is because fit indices are not only sensitive to misspecification but are also susceptible to various model, estimation, and data characteristics. As a solution, methodologists have proposed four principal approaches to obtain so-called tailored cutoffs, which are generated specifically for a given setting. Here, we review these approaches. We find that none of these approaches provides guidelines on which fit index (out of all fit indices of interest) is best suited for evaluating whether the model fits the data in the setting of interest. Therefore, we propose a novel approach combining a Monte Carlo simulation with receiver operating characteristic (ROC) analysis. This so-called simulation-cum-ROC approach generates tailored cutoffs and additionally identifies the most reliable fit indices in the setting of interest. We provide R code and a Shiny app for an easy implementation of the approach. No prior knowledge of Monte Carlo simulations or ROC analysis is needed to generate tailored cutoffs with the simulation-cum-ROC approach.

## Introduction

To test the goodness of confirmatory factor analysis (CFA) models—and structural equation models more generally—researchers routinely rely on model fit indices (Jackson et al., 2009; Kline, 2016). Next to the chi-square test of exact model fit (${\chi}^{2}$; e.g., Bollen, 1989)^1^, some of the most commonly used global fit indices are the comparative fit index (CFI; Bentler, 1990), the root mean square error of approximation (RMSEA; Steiger, 1990), and the standardized root mean residual (SRMR; Bentler, 1995). Those fit indices quantify model–data (mis-)fit in a continuous way. However, because fit indices are hard to interpret in isolation, researchers usually rely on cutoffs (or “thresholds”) for fit indices that enable them to make binary decisions about whether a model fits the data or not.

Methodologists commonly derive such cutoffs for fit indices from Monte Carlo simulation studies (for an overview and detailed description, see Boomsma, 2013). Such simulation studies examine how fit indices behave across controlled scenarios. Methodologists specify true data-generating (or population) models and determine misspecification of various forms (e.g., in loadings or number of factors) in the analysis model, the model to be tested. By repeatedly generating (i.e., simulating) random data from each population model and fitting the analysis model to each data, they obtain fit index distributions. A cutoff should then represent the fit index value that only rejects the analysis model if it is misspecified.

On the basis of such a simulation study, Hu and Bentler (1999) derived a set of cutoffs that have since become the de facto standard in the field. Their simulation study covered a limited set of scenarios assumed to represent typical empirical settings. More specifically, the scenarios always encompassed three-factor models with 15 items. Hu and Bentler specified those models to have varying item and factor distributions, drew samples of various sizes, and misspecified either loadings or factor covariances. Based on their investigation of those scenarios, Hu and Bentler proposed that CFI should be above or close to .950, RMSEA should be below or close to .060, and SRMR should be below or close to .080 to indicate good model fit.

In empirical applications, researchers compare their obtained fit index values against these cutoffs to evaluate whether their model fits the data (i.e., is assumed to be correctly specified) or not (i.e., is assumed to be misspecified). This simple binary (yes/no) decision-making on model fit using the same, fixed cutoffs across diverse empirical settings (oftentimes different from the initial simulation scenarios) has been common practice in research involving latent-variable models for decades (e.g., Jackson et al., 2009).

However, such fixed cutoffs for fit indices are more problematic than many researchers may realize. This is because fit indices are not only sensitive to misspecification, as intended, but undesirably susceptible to a range of model, estimation, and data characteristics. These characteristics include, for example, the loading magnitudes, the type of estimator, the sample size, and interactions thereof, especially when the model is misspecified (e.g., Groskurth et al., 2024; Heene et al., 2011; Moshagen & Auerswald, 2018; Shi et al., 2019; Xia & Yang, 2018, 2019; for an overview, see Niemand & Mai, 2018). Likewise, the (non-)normality of the multivariate response distribution influences fit indices, regardless of whether the model is correctly specified or misspecified (e.g., Fouladi, 2000; Yuan & Bentler, 1999, 2000b; Yuan et al., 2004). Further complicating matters, different fit indices react differently to model misspecifications, extraneous characteristics, and the interaction between them (Groskurth et al., 2024; Lai & Green, 2016; Moshagen & Auerswald, 2018).

The susceptibility of fit indices to such characteristics other than model misspecification leads to two key challenges in model evaluation. First, *the performance ability of fit indices to detect model misspecification can vary greatly across empirical settings.* Some fit indices react more strongly to misspecification than others in certain settings (and vice versa, e.g., Moshagen & Auerswald, 2018). This differential performance threatens the ability of fit indices to discriminate between correctly specified and misspecified models (e.g., Reußner, 2019). No fit index universally outperforms others (for an overview, see Groskurth et al., 2024; Niemand & Mai, 2018). Second, *cutoffs for fit indices pertain only to specific* *scenarios* (i.e., combinations of model, estimation, and data characteristics). Simulation studies can only cover a limited number of combinations of such characteristics. In empirical settings that diverge markedly from the simulation scenarios generating the cutoffs, these cutoffs may no longer allow for valid judgments of model fit (e.g., Hu & Bentler, 1999; McNeish & Wolf, 2023a).

It is impossible to arrive at general rules on the performance of specific fit indices, let alone fixed cutoffs that are universally applicable across settings. It is likewise impossible to devise a simulation study that includes all possible scenarios. Although Hu and Bentler (1999) already warned against overgeneralizing their cutoffs, their cautionary note seems to have been largely unheeded in applied research (e.g., Jackson et al., 2009; McNeish & Wolf, 2023a). In practice, researchers apply cutoffs for fit indices rather uncritically. Treating the once-proposed cutoffs and sets of fit indices as “golden rules” can result in erroneous conclusions regarding model fit (Marsh et al., 2004; for examples, see McNeish & Wolf, 2023a). Such erroneous results threaten the integrity of scientific findings.

A solution that has long been proposed is to use tailored cutoffs for fit indices customized to a specific setting of interest (Millsap, 2013; see also Kim & Millsap, 2014, based on Bollen & Stine, 1992). Tailored cutoffs are not yet widely used despite recently regaining traction (e.g., McNeish & Wolf, 2023a, b). Toward the ultimate aim of helping researchers transition to more valid model evaluation practices via tailored cutoffs, the first goal of this article was to review and summarize existing approaches to generating tailored cutoffs. Such a systematic overview is missing from the current literature. As this review will reveal, existing approaches to generating tailored cutoffs have unique strengths and, while generally superior to fixed cutoffs, share some limitations. Chief among these limitations is that none of the existing approaches allows an evaluation of the differential performance of fit indices. They provide no guidelines on which fit index (out of all fit indices of interest) reacts most strongly to misspecification and, thus, best discriminates between correctly specified and misspecified models in a given setting.

Therefore, the second goal of our article was to introduce a novel approach that builds on—and extends—prior approaches (e.g., McNeish & Wolf, 2023a, b; Millsap, 2013; Pornprasertmanit, 2014). It combines a Monte Carlo simulation, an often-used procedure in psychometrics, with a receiver operating characteristic (ROC) analysis. Our so-called simulation-cum-ROC approach answers two questions: (1) Which fit indices, if any, perform well (or even best) in a setting of interest? (2) Which cutoffs best discriminate between correctly specified and misspecified models in that setting? In this regard, our approach generates tailored cutoffs for well-performing fit indices, whereas the best-performing fit index is considered the most decisive for model evaluation. We illustrate this approach with empirical examples and provide complete R code as well as a Shiny app that facilitates its application.

### The logic behind generating cutoffs for fit indices

In recent years, methodologists have advocated moving away from using fixed cutoffs and proposed several approaches to generate cutoffs tailored to the empirical setting of interest (e.g., McNeish & Wolf, 2023a; Millsap, 2013; Pornprasertmanit, 2014). Before introducing any of these approaches to tailored cutoffs, we must highlight two important distinctions foundational to generating cutoffs for fit indices, whether fixed or tailored. The first distinction is between the analysis model (i.e., the latent-variable model one seeks to test) and the population model (i.e., the true model that generated the data). The second distinction is between empirical settings and hypothetical scenarios. In an empirical setting (i.e., fitting the analysis model to empirical data to test its fit), one never knows whether the analysis model is correctly specified or misspecified because the true data-generating mechanism (i.e., the population model) is always unknown. By contrast, in a hypothetical scenario (which can be used for simulating data), one knows whether the analysis model is correctly specified or misspecified because one can define both the analysis model and the population model that generates the data. These distinctions between analysis and population models, as well as between empirical settings and hypothetical scenarios, are crucial for all approaches generating cutoffs for fit indices.

It is also pertinent to all approaches to define different hypotheses about how the empirical data might have been generated. Researchers usually follow the Neyman–Pearson approach to hypothesis testing (Neyman & Pearson, 1928, 1933; see Biau et al., 2010; Moshagen & Erdfelder, 2016; Perezgonzalez, 2015). The Neyman–Pearson approach requires specifying a null hypothesis (*H*_*0*_) and an alternative hypothesis (*H*_*1*_). *H*_*0*_ states that a population model identical (or nearly identical) to the analysis model has generated the data; the analysis model captures all relevant features of the population model. It is correctly specified. *H*_*1*_ states that an alternative population model different from the analysis model has generated the data; the analysis model is underspecified (i.e., misspecified) compared to the population model to an intolerable degree and fails to capture its relevant features. It is misspecified.

Cutoffs for fit indices, in essence, are needed to discriminate between *H*_*0*_ and *H*_*1*_ in empirical settings where the population model is unknown. However, one cannot generate cutoffs in an empirical setting where the population model is unknown; one needs to generate cutoffs in a hypothetical scenario where the population model is known.

The general procedure to derive either fixed or tailored cutoffs is as follows: Fit index distributions for correctly specified (*H*_*0*_) and misspecified (*H*_*1*_) analysis models are derived. The goal is to choose a cutoff (e.g., corresponding to a certain percentile from the fit index distributions) that accurately classifies correctly specified models as correctly specified and misspecified models as misspecified. The chosen cutoff should minimize the misclassification of correctly specified models as misspecified (type I error rate) and of misspecified models as correctly specified (type II error rate).

Fixed cutoffs are generated to broadly cover a generic set of hypothetical scenarios that are assumed to occur regularly in empirical settings (e.g., three-factor models with 15 items in the case of Hu and Bentler, 1999). Once created, researchers use this single set of cutoffs across diverse empirical settings. In contrast, tailored cutoff approaches define the hypothetical scenario closely to the empirical setting of interest, such as using the same sample size of the empirical data and the same analysis model of interest. Each time researchers consider a new empirical setting, they must derive a new set of tailored cutoffs.

Once (either fixed or tailored) cutoffs are derived from hypothetical scenarios with known population models, one then uses these cutoffs to test which hypothesis—*H*_*0*_ or *H*_*1*_—is more plausible for their analysis model fit to empirical data generated from an unknown population model. If empirical fit index values pass their cutoffs, one accepts the analysis model. Accepting the analysis model means that *H*_*0*_ seems more plausible than *H*_*1,*_ given the empirical data. If empirical fit index values fail their cutoffs, one rejects the analysis model. Rejecting the analysis model means that *H*_*1*_ seems more plausible than *H*_*0,*_ given the empirical data. Whether *H*_*0*_ or *H*_*1*_ is indeed true will be left unanswered as the population model generating the empirical data always remains unknown (Neyman & Pearson, 1928).^2^

### A review of existing approaches to generating tailored cutoffs

Whereas fixed cutoffs are usually derived once in a single simulation study, covering a range of scenarios, various approaches have been specified to derive cutoffs tailored to the specific empirical setting at hand. Currently, there are four principal approaches to generating tailored cutoffs (Table 1)^3^ that fall on a continuum from parametric to non-parametric:

1. The ${\chi}^{2}$ distribution-based approach generates cutoffs by relying on statistical assumptions of the ${\chi}^{2}$ distribution without and with misspecification (Moshagen & Erdfelder, 2016).
2. The regression-based approach generates cutoffs based on meta-regression results from a prior simulation study (Nye & Drasgow, 2011; Groskurth et al., 2024). The regressions predict cutoffs from various model, estimation, and data characteristics, allowing the researcher to account for characteristics that influence fit indices.
3. The dynamic simulation approach generates cutoffs based on fit index distributions from an analysis model fit to multiple samples from known population models (McNeish & Wolf, 2023a, b; Millsap, 2007, 2013; Mai et al., 2021; Niemand & Mai, 2018; Pornprasertmanit, 2014).
4. The bootstrap approach generates cutoffs based on fit index distributions by fitting the analysis model to resampled empirical data transformed as if the analysis model does (or does not) fit it (Bollen & Stine, 1992; Kim & Millsap, 2014).

**Table 1:** Existing approaches to generate tailored cutoffs

Principal approach	Author(s)	Type I error?	Type II error?	Performance of fit indices?	Tailored to …	Helpful resources
**χ**^**2**^** Distribution**: Generating cutoffs based on distributional assumptions	Moshagen & Erdfelder (2016)	✓	✓	✗	Sample size, degrees of freedom, and number of items for fit indices, which distributions can be derived from ${\chi}^{2}$	Shiny app: https://sempower.shinyapps.io/sempower, https://sjak.shinyapps.io/power4SEM/ (Jak et al., 2021) R package: semPower (Moshagen & Bader, 2024) Tutorial: Jobst et al. (2023)
**Regression**: Generating cutoffs based on meta-regressions	Nye & Drasgow (2011)	✓	✗	✗	Sample size and response distribution for RMSEA and SRMR	Regression equation: included in the article
	Groskurth et al. (2024)	✓	✗	✗	Estimator, number of items, number of response options, response distribution, loading magnitude, sample size, and factor correlation for ${\chi}^{2}$, ${\chi}^{2}$/degrees of freedom*,* CFI, RMSEA, SRMR	Regression equation: included in the article R code: included in the article
**Dynamic simulation**: Generating cutoffs based on fit index distributions generated via a Monte Carlo simulation	Niemand & Mai (2018), Mai et al. (2021)	✓	✗	✗	All model, estimation, and data characteristics for available fit indices	R package: FCO (Niemand & Mai, 2025)
	Millsap (2007, 2013)	✓	✗	✗	All model, estimation, and data characteristics for available fit indices	R package: simsem (Pornprasertmanit et al., 2021), ezCutoffs (Schmalbach et al., 2019)
	McNeish & Wolf (2023a, b)	✓	✓	✗	All model, estimation, and data characteristics for available fit indices	Shiny app: https://dynamicfit.app/__landing__/ R package: dynamic (Wolf & McNeish, 2022) Mplus code: included in the article
	Pornprasertmanit (2014)	✓	✓	✗	All model, estimation, and data characteristics for available fit indices	
**Bootstrap**: Generating cutoffs based on bootstrapped fit index distributions	Bollen & Stine (1992), Kim & Millsap (2014)	✓	✗	✗	All model, estimation, and data characteristics for available fit indices	R package: simsem (Pornprasertmanit et al., 2021), lavaan (Rosseel, 2012) R code: included in the article
	Yuan & Hayashi (2003), Yuan et al. (2004, 2007)	✓	✓	✗	All model, estimation, and data characteristics for available fit indices	R package: lavaan (Rosseel, 2012)
Dynamic simulation + ROC analysis	Present article (simulation-cum-ROC)	✓	✓	✓	All model, estimation, and data characteristics for available fit indices	Shiny app: https://kg11.shinyapps.io/tailoredcutoffs/ R code: included in Additional File 1 of the Supplementary Online Material

*Note*. Mai et al. (2021) provided general recommendations on the performance of fit indices depending on the purpose of the research question (testing an established versus a novel model), the focus of estimation (testing a measurement model or structural model), and sample size (below or above *N* = 200) derived from an extensive simulation study. As those recommendations are based on a prior simulation study instead of being specifically derived for the setting of interest, we did not highlight them in this table

### ${\chi}^{2}$ distribution-based approach

One option to generate tailored cutoffs is via the parametric ${\chi}^{2}$ distribution-based approach as outlined by Moshagen and Erdfelder (2016, which seems to be partly based on MacCallum et al., 1996; see also Jak et al., 2021; Jobst et al., 2023). The core idea of the ${\chi}^{2}$ distribution-based approach to tailored cutoffs is to infer the distributions of correctly specified and misspecified models from the known central and non-central ${\chi}^{2}$ distributions. The central and non-central ${\chi}^{2}$ distributions can then be used to determine cutoffs. This works both for the ${\chi}^{2}$ test statistic itself and for fit indices that incorporate it (such as RMSEA).

The approach rests on the assumption that the ${\chi}^{2}$ test statistic follows a central ${\chi}^{2}$ distribution if the analysis model is correctly specified—but a non-central ${\chi}^{2}$ distribution if the analysis model is misspecified. A non-centrality parameter determines how much the non-central ${\chi}^{2}$ distribution deviates from the central ${\chi}^{2}$ distribution. Crucially, this non-centrality parameter depends on the misspecification of the analysis model and the sample size (for a detailed description, see Bollen, 1989; Chun & Shapiro, 2009; Moshagen & Erdfelder, 2016).

To derive tailored cutoffs, users define an effect size difference (i.e., some degree of intolerable misspecification based on the non-centrality parameter) between the central and non-central ${\chi}^{2}$ distribution. The expected value of the central ${\chi}^{2}$ distribution equals the degrees of freedom of the analysis model of interest. It is the distribution for the ${\chi}^{2}$ test statistic given that the analysis model is correctly specified. The expected value of the non-central ${\chi}^{2}$ distribution equals the degrees of freedom of the analysis model of interest plus the effect size (i.e., the intolerable degree of misspecification defined by the non-centrality parameter). It is the distribution for the ${\chi}^{2}$ test statistic given that the analysis model is misspecified. Those two distributions allow users to derive a cutoff for the ${\chi}^{2}$ test statistic at a specific ratio of type I and type II error rates. Typically, the type I and type II error rates are balanced (i.e., equally small).

The ${\chi}^{2}$ distribution-based approach has the advantage of computational speed. Statistical tools such as R rapidly solve the equations needed to generate cutoffs. However, a disadvantage of this procedure is the limited extent of tailoring. The approach can only generate cutoffs for fit indices that are transformations of the ${\chi}^{2}$ test statistic (e.g., RMSEA). It is not applicable to fit indices that are based, for example, on standardized residuals (e.g., SRMR) and, thus, do not follow a known distribution. Moreover, users can only calculate tailored cutoffs from Moshagen and Erdfelder’s (2016) ${\chi}^{2}$ distribution-based approach under the assumption that items follow a multivariate normal distribution, in which case the ${\chi}^{2}$ distribution is known. Non-normal multivariate distributions of the items (e.g., Fouladi, 2000; Yuan & Bentler, 1999, 2000b; Yuan et al., 2004) or large models with many items (Moshagen, 2012) violate the distributional assumptions of the ${\chi}^{2}$ test statistic. Different test statistics (e.g., Yuan & Bentler, 2007) are necessary to generate valid cutoffs that are not always straightforward to handle. In sum, the ${\chi}^{2}$ distribution-based approach limits the extent to which users can tailor cutoffs to their specific setting of interest and the range of fit indices for which users can generate the cutoffs (see Table 1).

### Regression-based approach

Another option to generate tailored cutoffs is via the parametric regression-based approach. The basic idea is to predict tailored cutoffs for a given empirical setting using a regression formula (e.g., Groskurth et al., 2024; Nye & Drasgow, 2011). This enables users to account for at least some of the characteristics of their empirical setting when choosing appropriate cutoffs.

The regression-based approach underlies a single, although typically very extensive, simulation study in the background. This simulation ideally covers many different model, estimation, and data characteristics (e.g., one- versus two-factor models, different numbers of items, and different distributions of the items). Cutoffs at certain type I or type II error rates are derived for each of the different scenarios. Cutoffs are considered dependent variables and regressed on the model, estimation, and data characteristics considered in the simulation. The regression formula thus comprises predictors with associated regression weights that contain information about how the various model, estimation, and data characteristics covered in the simulation (e.g., number of items, type of estimator, and distribution of responses) influence cutoffs at certain type I or type II error rates. To derive tailored cutoffs, users simply plug their model, estimation, and data characteristics of their setting of interest into the formula.

Hence, users predict tailored cutoffs by using a regression formula from a large, ideally extensive simulation study. Although the simulation study underlying the regression formula should be extensive, it does not necessarily cover a scenario similar to the empirical setting of interest. However, the formula allows for extrapolation; thus, it allows for the prediction of cutoffs for settings not covered in the initial simulation scenarios. Although extrapolation is only advisable for empirical settings that do not diverge strongly from the scenarios in the initial simulation, it helps to tailor cutoffs to a wider variety of settings than initially covered by the simulation scenarios.

Nye and Drasgow (2011) and Groskurth et al. (2024) followed the regression-based approach. Nye and Drasgow (2011) provided regression formulae for RMSEA and SRMR. Besides the cutoffs, they considered the response distribution, the sample size, and the type I error rate in the formulae. Their models had two factors, 15 items, and they estimated them with diagonally weighted least squares. Groskurth et al. (2024) considered more fit indices and a much wider range of characteristics: They provided regression formulae for ${\chi}^{2}$, ${\chi}^{2}$/degrees of freedom*,* CFI, RMSEA, and SRMR. Estimators, number of items, response distributions, response options, loading magnitudes, sample size, and number of factors served as predictors in the formulae.

Similar to the ${\chi}^{2}$ distribution-based approach, the regression-based approach has the advantage of speed. Users merely have to plug the characteristics of their empirical setting into the formula, commonly solved by a statistical tool such as R. However, the regression formula is only as inclusive as the simulation study from which it was derived—although extrapolation is possible for settings different from the initial simulation scenarios. Further, users can only obtain cutoffs for those fit indices that are considered in the simulation study from which the regression formula hails. Akin to the ${\chi}^{2}$ distribution-based approach, the regression-based approach limits the extent to which users can tailor cutoffs to their specific empirical setting and the range of fit indices (see Table 1).

### Dynamic simulation approach

A third approach that allows for a much greater extent of tailoring cutoffs is what we call the “dynamic” simulation approach (following McNeish & Wolf, 2023a). Like the fixed cutoff approach (but also like the regression-based approach for tailored cutoffs), the dynamic approach uses Monte Carlo simulations to generate cutoffs. Crucially, however, the simulations are performed *for the specific empirical setting at hand* on a case-by-case basis—instead of relying on generic simulation results (McNeish & Wolf, 2023a, b; Millsap, 2007, 2013; Mai et al., 2021; Niemand & Mai, 2018; Pornprasertmanit, 2014; for nested models, see Pornprasertmanit et al., 2013).

Simulation scenarios are well known (dating back to the initial Hu & Bentler, 1999, article); we describe them in detail here to enable users to apply the dynamic simulation approach: Users need to define a population model, simulate data (i.e., draw multiple samples) from that population model, and fit an analysis model to the simulated data. The analysis model is identical (or nearly identical) to the population model; it captures all relevant features of the population model and is, thus, correctly specified. After fitting the analysis model to the data, users record the fit index values of each analysis model. A cutoff can then be set based on a specific percentile, commonly the 95th or 90th, of the resulting fit index distribution (equivalently, on the 5th or 10th for fit indices where higher values indicate better fit). At this percentile, the cutoff categorizes 95% or 90% of correctly specified models as correctly specified and 5% or 10% of correctly specified models as misspecified (i.e., the type I error rate).

Users may repeat the procedure with the same analysis model but a population model with more parameters than the analysis model. As such, one fixes non-zero parameters in the population model to zero in the analysis model (e.g., Hu & Bentler, 1998). The analysis model is, thus, underspecified (i.e., misspecified) relative to the population model; it fails to capture relevant features of the population model. Including a misspecified scenario allows for evaluating how many misspecified models a cutoff categorizes as correctly specified (i.e., the type II error rate).

Crucially, to arrive at cutoffs *tailored* to the setting of interest, the analysis and population models are not just any models but are chosen to match the given empirical setting. Each time users assess a new empirical setting (i.e., different model, estimation, and data characteristics), they must derive a new set of cutoffs via Monte Carlo simulations. This makes the approach dynamic and distinguishes it from approaches that rely on generic simulation studies (i.e., most prominently, the fixed cutoff approach). Thus, the dynamic simulation approach eliminates the problem that empirical settings may deviate from scenarios underlying the cutoffs by specifying the simulation scenario just like the empirical setting of interest.

The dynamic simulation approach is computationally intensive, more intensive than the χ^2^ distribution-based and regression-based approaches, because a simulation study has to be run anew for every setting of interest. For the same reason, it has the advantage of being very flexible. It generates tailored cutoffs for *all* fit indices available in a given statistical program to the specific model, estimation, and data characteristics of the analysis setting at hand (see Table 1). Combined with computers’ continuously increasing statistical power, this is one of the reasons why this approach has recently gained traction (McNeish & Wolf, 2023a, b).

### Bootstrap approach

Cutoffs tailored to the given analysis setting at hand can not only be generated via (dynamic) Monte Carlo simulations, which are essentially parametric bootstrap approaches (simulating, i.e., resampling, data based on model parameters), but also via non-parametric bootstrap approaches (i.e., resampling observed data). A fourth approach uses such non-parametric bootstrapping to generate tailored cutoffs from empirical data transformed as if the analysis model does (or does not) fit it (Bollen & Stine, 1992; Kim & Millsap, 2014; Yuan & Hayashi, 2003; Yuan et al., 2004, 2007).

In the following, we illustrate Bollen and Stine’s (1992) and Kim and Millsap’s (2014) bootstrap approach in more detail. The bootstrap approach transforms each observation in the empirical data using the data-based and model-implied covariance and mean structure (see also Yung & Bentler, 1996). After the transformation, users obtain data that behaves as if the analysis model had generated it. The algorithm resamples the transformed data (with replacement), fits the analysis model to each resampled data, and records the values of fit indices for each. The bootstrap approach outlined above allows evaluating type I error rates (i.e., incorrectly rejecting a correctly specified model) for cutoffs that correspond to a certain percentile of the resulting fit index distributions. Yuan and Hayashi (2003), as well as Yuan et al. (2004, 2007), developed an extended bootstrap approach that also allows investigating power (i.e., correctly rejecting a misspecified model—the complement of the type II error rate).

The bootstrap approach is very flexible, similar to the dynamic simulation approach (see Table 1). Through repeated resampling, users can generate cutoffs for all available fit indices tailored to all choice characteristics. This comes at the expense of greater computational intensity than the ${\chi}^{2}$ distribution-based and regression-based approaches.

## Limitations of the existing approaches

All four approaches of generating tailored cutoffs have their merits and constitute a clear advancement over fixed cutoffs, allowing for more valid cutoffs that control type I and/or type II errors. Some approaches have an advantage in terms of computational speed in arriving at tailored cutoffs (i.e., the ${\chi}^{2}$ distribution-based and regression-based approaches, both parametric). Others stand out as they are very general and generate cutoffs for a wide range of fit indices across a wide range of characteristics (i.e., the parametric dynamic simulation approach and the non-parametric bootstrap approach).

However, these approaches also have specific limitations (see Table 1). One limitation they share is that they do not assess which fit index (among several fit indices a researcher may consider) reacts most strongly to misspecification. Knowing which fit index is, thus, best able to discern correctly specified from misspecified models in the setting of interest would guide researchers on which fit indices they should rely on for judging model fit. Such guidance on how much weight to assign to each fit index is especially needed when fit index decisions on model fit disagree, which often occurs in practice (e.g., Lai & Green, 2016; Moshagen & Auerswald, 2018).

We, therefore, introduce an approach that builds on previous approaches and extends them by (1) identifying well-performing (and best-performing) fit indices in a specific setting of interest while (2) generating tailored cutoffs that balance both type I and type II error rates. This new approach is both general and adaptable enough to support valid judgments of model fit across various settings that researchers may encounter.

### A novel approach to tailored cutoffs: The simulation-cum-ROC approach

Our so-called simulation-cum-ROC approach augments the dynamic simulation approach (e.g., McNeish & Wolf, 2023a; Millsap, 2013; Pornprasertmanit, 2014) that is currently gaining traction among applied researchers and builds on a long tradition of generating cutoffs through Monte Carlo simulations (dating back to the initial Hu & Bentler, 1999, article). The unique contribution of our approach is combining the dynamic simulation approach with receiver operating characteristic (ROC) analysis. The ROC analysis enables us to (1) rank the performance of any fit index in the setting of interest, including—but not limited to—the canonical fit indices on which we focus in this article (i.e., ${\chi}^{2}$, CFI, RMSEA, SRMR). Further, the dynamic simulation approach, in combination with ROC analysis, enables us to (2) generate tailored cutoffs at balanced type I and type II error rates for well-performing fit indices. Our approach thus allows for a more informative and rigorous evaluation of model fit.

**Fig. 1:**
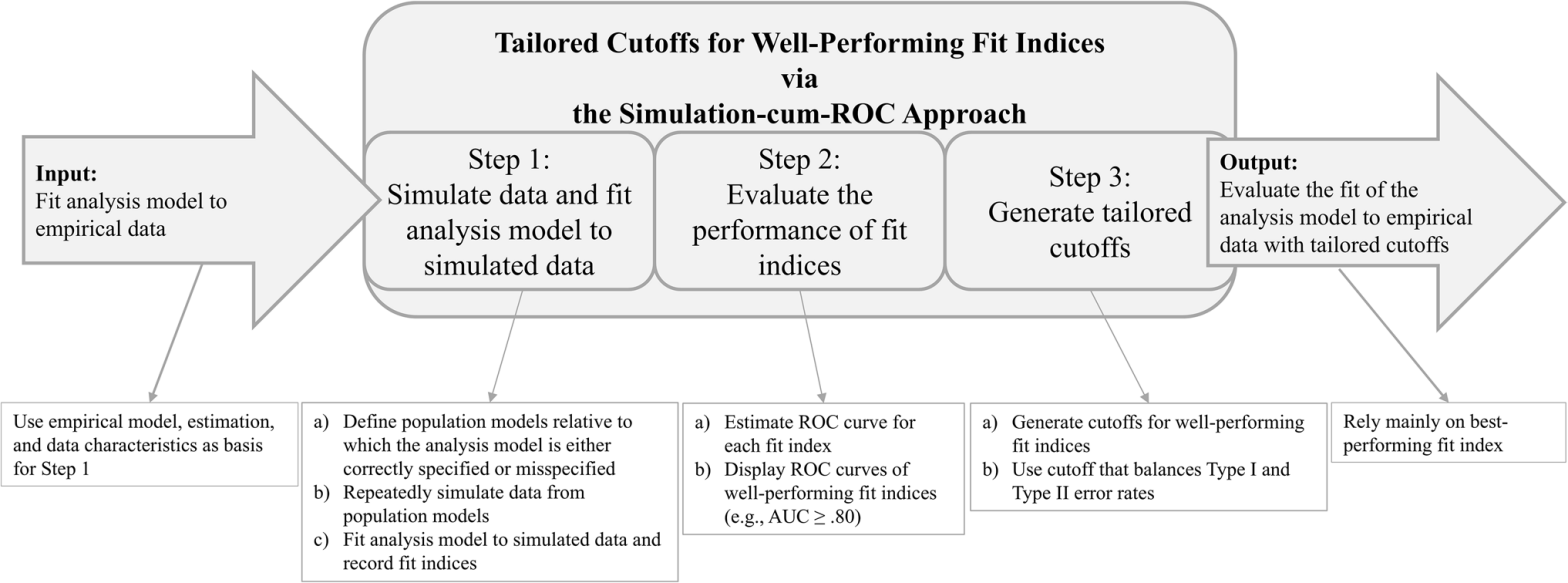
The simulation-cum-ROC approach

In a nutshell, the simulation-cum-ROC approach works as follows. First, we use a Monte Carlo simulation to generate data from two population models, each representing different assumptions about the true data-generating mechanism. One population model is structurally identical to the analysis model one seeks to test, such that the analysis model is correctly specified relative to the population model (*H*_*0*_). The other population model diverges from that analysis model, such that the analysis model is misspecified relative to the population model (*H*_*1*_). We fit the analysis model to data simulated from the two population models and record the fit index values. Those simulations are conducted for the empirical setting of interest and, thus, resemble what is done in other dynamic simulation approaches (e.g., McNeish & Wolf, 2023a; Millsap, 2013; Pornprasertmanit, 2014). Second, as a new feature, we analyze the fit index distributions with ROC analysis in addition to what is done in dynamic simulation approaches. ROC analysis equips researchers with a tool to rank fit indices in terms of their ability to discriminate between correctly specified and misspecified models. Third, we generate cutoffs not for all fit indices of interest but only for those that appear well-performing in the given scenario—these cutoffs balance type I and type II error rates. We visualized the three steps to generate tailored cutoffs for well-performing fit indices in Fig. 1.

### Fundamentals of ROC analysis

Before outlining the details of our simulation-cum-ROC approach, we briefly introduce ROC analysis. We base the introduction of ROC analysis on Flach (2016) and Padgett and Morgan (2021). Flach (2016) provided a general description of ROC analysis, and Padgett and Morgan (2021) connected ROC analysis to model fit evaluation.

ROC analysis originated within the context of signal detection theory in communication technology (for a detailed overview of the history of ROC analysis and signal detection theory, see Wixted, 2020). It provides a tool to evaluate the ability of a binary classifier to make correct diagnostic decisions in diverse scenarios, such as hypothesis testing. ROC analysis finds the optimal value for a classifier in making a diagnostic decision, such as classifying an analysis model as correctly specified or misspecified. It has supported decision-making in medicine for many decades and gained popularity in machine learning (for an overview, see Majnik & Bosnić, 2013).

Fit indices are essentially continuous classifiers that typically indicate better fit for correctly specified models and poorer fit for misspecified models. The cutoffs for these fit indices serve as decision thresholds. These cutoffs should be selected to maximize the share of analysis models that are classified as either correctly specified or misspecified.

Cutoffs for fit indices have a high sensitivity (i.e., true-positive rate) if they classify a high share of misspecified models as misspecified (i.e., true positive) and only a small share of misspecified models as correctly specified (i.e., false-negative, type II error). In turn, cutoffs for fit indices have a high specificity (i.e., true-negative rate) if they classify a high share of correctly specified models as correctly specified (i.e., true negative) and only a small share of correctly specified models as misspecified (i.e., false positive, type I error). The formulae to calculate sensitivity and specificity read as

1 $$Sensitivity\, \left(or\;true-positive\;rate \right)=\frac{Number\ of\ True\ Positives}{Number\ of\ True\ Positives + Number\ of\ False\ Negatives}$$

2 $$Specificity\,\left(or\ true-negative\ rate\right)= \frac{Number\ of\ True\ Negatives}{Number\ of\ True\ Negatives + Number\ of\ False\ Positives}$$

The goal is to find a cutoff for each fit index that provides an optimal balance between sensitivity and specificity (i.e., which maximizes the sum of sensitivity and specificity – 1, i.e., the Youden index). Such an optimal cutoff has a high accuracy, which means that the share of true positives and true negatives is large among all classified cases (i.e., the total number of converged models in simulation runs):

3 $$Accuracy=\frac{Number\ of\ True\ Positives\ + Number\ of\ True\ Negatives}{Number\ of\ True\ Positives + Number\ of\ True\ Negatives + Number\ of\ False\ Positives + Number\ of\ False\ Negatives}$$

**Fig. 2:**
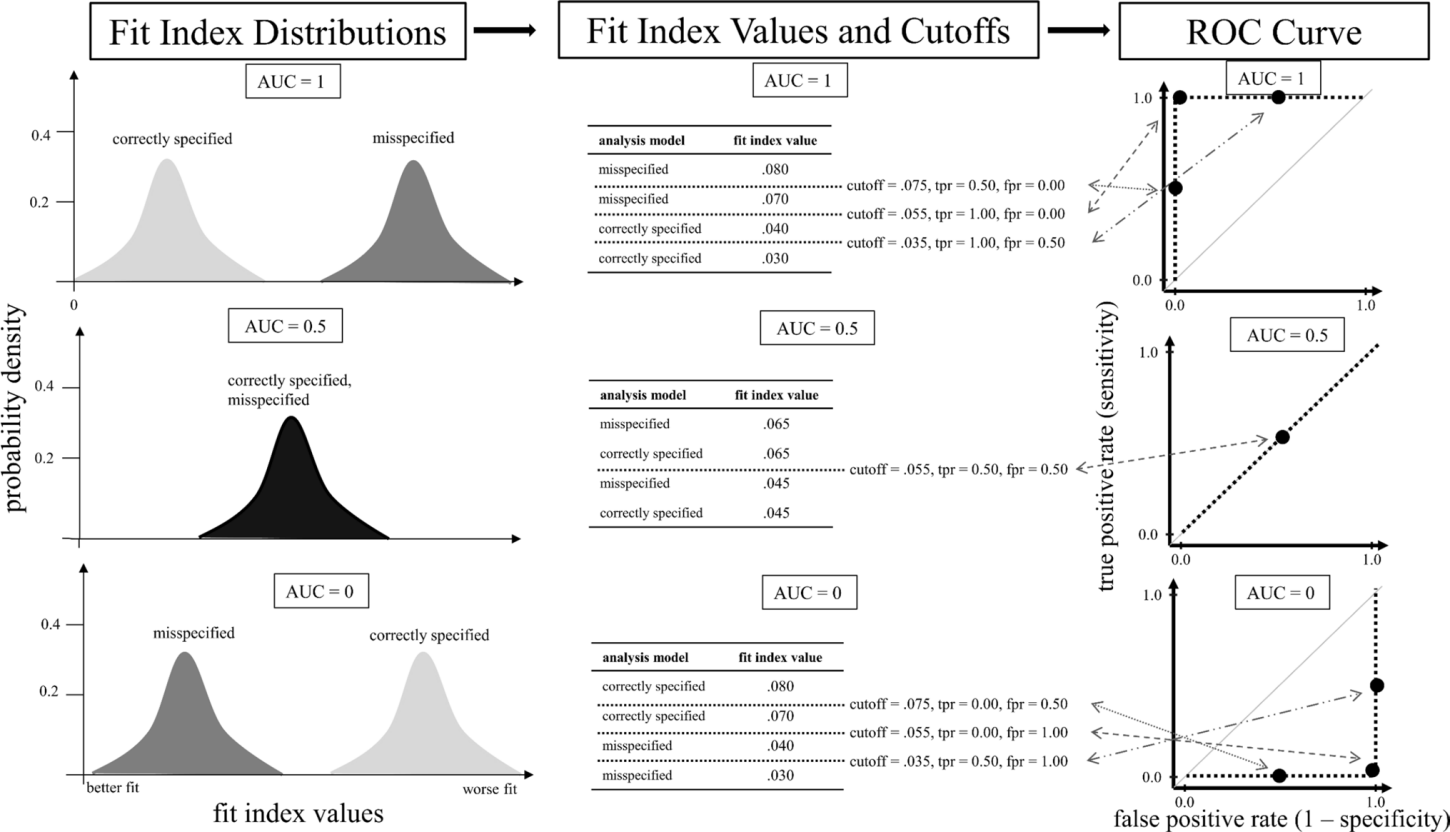
Relation of fit index distributions, cutoffs, and the ROC curve for different AUCs. *Note.* The figure shows fit index distributions and a sample of their fit index values. It further includes true- and false-positive rates of cutoffs estimated from the sample of fit index values. The ROC curve visualizes the true- and false-positive rates of cutoffs. The interplay of fit index distributions, true- and false-positive rates, and the ROC curve differ across AUCs. tpr = true-positive rate; fpr = false-positive rate

An ROC curve visualizes the sensitivity and specificity at different cutoffs. These cutoffs may be generated arbitrarily (within the range of fit index values, e.g., Flach, 2016), or the actual fit index values are taken as cutoffs (as done here, following Thiele & Hirschfeld, 2021). The graph visualizing the ROC curve has sensitivity (or true-positive rate) on its *Y*-axis and 1 − specificity (or false-positive rate) on its *X*-axis. The area under the curve (AUC) quantifies the information of the ROC curve. We visualized the relationship between the distributions of a fit index, true- and false-positive rates of cutoffs, ROC curve, and AUC values in Fig. 2.

The AUC ranges between 0 and 1. It indicates the discrimination ability of a fit index at different cutoffs. An AUC of 1 is most favorable; it implies that all cutoffs have a true-positive rate of 1 or a false-positive rate of 0. Thus, 100% of the time, the fit index will correctly discriminate between correctly specified and misspecified models (e.g., D’Agostino et al., 2013). The optimal cutoff, with the optimal balance between sensitivity and specificity, has a true-positive rate of 1 and a false-positive rate of 0—the ROC curve peaks in the upper left of the graph. Fit index distributions from correctly specified and misspecified models do not overlap (see Fig. 2).

An AUC of 0.5 can imply different things, but most importantly, it can imply that all cutoffs have equal true- and false-positive rates. The discrimination ability of the fit index at different cutoffs is no better than a guess (e.g., D’Agostino et al., 2013). No optimal cutoff can be identified—the ROC curve is an ascending diagonal. Fit index distributions from correctly specified and misspecified models completely overlap; no distinction is possible (see Fig. 2).

An AUC of 0 implies that all cutoffs have a true-positive rate of 0 or a false-positive rate of 1. The fit index has no discrimination ability at all at different cutoffs. An optimal cutoff cannot be identified—the ROC curve peaks in the lower right of the graph. Fit index distributions do not overlap; however, fit index values from correctly specified models behave unexpectedly and indicate worse fit than those from misspecified models (see Fig. 2).

Overall, the outlined relations indicate that the AUC quantifies what the ROC curve visualizes, namely, the performance of fit indices in terms of true- and false-positive rates at different cutoffs. The optimal cutoff is the one that has the highest sum of sensitivity (i.e., true-positive rate) and specificity (i.e., 1 – false-positive rate) across all evaluated cutoffs. Thus, the optimal cutoff shows up as a peak in the upper left of the graph (i.e., highest true-positive rate and lowest false-positive rate).

### Combining Monte Carlo simulation with ROC analysis to generate tailored cutoffs for fit indices

Having reviewed the basics of ROC analysis, we now detail our simulation-cum-ROC approach to (1) evaluate the performance of fit indices and (2) generate tailored cutoffs. We walk the reader through each step of the procedure shown in Fig. 1.

### Input: Fit analysis model to empirical data

Suppose we want to test whether a six-item scale measures a single underlying factor as its theory proposes. Survey data, including 500 participants’ responses to the six items of the scale, forms the basis of our empirical setting. We fit our analysis model—a one-factor CFA model—to the empirical data using robust maximum likelihood (MLR)^4^ estimation.

We aim to test two hypotheses. *H*_*0*_ states that a population model identical (or nearly identical) to the analysis model (i.e., a one-factor model) has generated the empirical data; the analysis model captures all relevant features of the population model. If empirical evidence favors *H*_*0*_, we want to accept this analysis model. *H*_*1*_ states that an alternative population model different from the analysis model has generated the empirical data; the analysis model is misspecified compared to the population model to an intolerable degree and fails to capture its relevant features. If empirical evidence favors *H*_*1*_, we want to reject the analysis model. Thus, we define two diverging states of the world that describe how the empirical data may have been generated (i.e., *H*_*0*_ and *H*_*1*_), and we can find evidence in favor of one or the other.

To test the two hypotheses, we obtain empirical fit index values fitting the analysis model to empirical data and compare those against cutoffs tailored to the specific characteristics of our empirical setting. We generate these cutoffs through the following three steps.

### Step 1: Simulate data and fit analysis model to simulated data

In the first step, we conduct a Monte Carlo simulation closely designed to mimic the real empirical setting in terms of the model of interest (e.g., number of items, loading magnitudes), the analytical strategy (e.g., MLR estimator), and the data characteristics (e.g., *N* = 500, multivariate distribution). The simulation-cum-ROC approach shares this basic idea with other dynamic simulation approaches (e.g., McNeish & Wolf, 2023a, b).

More specifically, following the Neyman–Pearson approach, we operationalize the two competing hypotheses, *H*_*0*_ versus *H*_*1*_, about the population model that may have generated the empirical data in the setting of interest through a Monte Carlo simulation scenario. Thereby, researchers need to define the *H*_*0*_ and *H*_*1*_ population models (e.g., Millsap, 2007, 2013; cf. McNeish & Wolf, 2023a, b). Whereas the *H*_*0*_ population model oftentimes simply equals the analysis model, the *H*_*1*_ population model is harder to define; researchers need to specify a certain degree of intolerable misspecification of the analysis model compared to the *H*_*1*_ population model.

As *H*_*1*_ population models are hard to define, they are usually predefined in dynamic simulation approaches (e.g., McNeish & Wolf, 2023a, b) and, thus, hidden from the researcher. For example, McNeish and Wolf (2023a, b) always use the same *H*_*1*_ population model in terms of model structure and additional parameters for all analysis models of the same type (e.g., one-factor CFA models).

We decided not to predefine the *H*_*1*_ population model but leave the definition of the *H*_*1*_ population model to the researcher (e.g., Millsap, 2007, 2013). To aid researchers in defining *H*_*1*_ population models, we provide guidance on defining the form and quantifying the degree of misspecification in the Discussion section. This guidance should make the definition of *H*_*1*_ population models more comparable and, thus, objective.

In our view, having the researcher explicitly specify the *H*_*1*_ population model is favorable to relying on implicit ones. It makes assumptions about the *H*_*1*_ population model transparent; researchers need to think about and justify their definition of intolerable misspecification. It is very important that researchers transparently outline their choices and provide a strong rationale for their hypotheses and models. Providing a strong rationale aligns with recent calls for more rigorous theory testing in psychology, formalized theories, and preregistration (e.g., Borsboom et al., 2021; Fried, 2020; Guest & Martin, 2021).

After defining the population models, we simulate data from the *H*_*0*_ population model structurally identical to the analysis model of interest (i.e., a one-factor CFA model). We also simulate data from the *H*_*1*_ population model that diverges substantially from the analysis model. For example, an *H*_*1*_ population model could have two factors, whereas the analysis model of interest has one factor. Notably, model are usually nested but they do not necessarly need to be nested (i.e., analysis and population models alike do not need to represent a subspace of each other), meaning that our approach is flexible regarding model definition.^5^^,^^6^

After repeatedly simulating data from the *H*_*0*_ and *H*_*1*_ population models (e.g., 500 times each), we fit the one-factor analysis model to all simulated data and record the values of the fit indices. We obtain distributions of fit index values for correctly specified models (under *H*_*0*_) and misspecified models (under *H*_*1*_).

### Step 2: Evaluate the performance of fit indices

After simulating data and obtaining fit index distributions, we evaluate and rank the performance of fit indices on the simulated data via the ROC curve and the AUC in particular, which is a unique feature of the simulation-cum-ROC approach. Both the ROC curve and the AUC reflect the balance of a fit index between the true-positive rate, or sensitivity, and the false positive rate, or 1 – specificity, at different potential cutoffs. The closer the fit index’s AUC is to 1, the higher its sensitivity and specificity across different potential cutoffs. Thus, the AUC quantifies how well a fit index discriminates between correctly specified and misspecified models—as such, we can rank fit indices according to their ability to detect misspecification in the specific scenario. Hence, the AUC provides guidance regarding which fit index (or indices) is best to judge the model’s fit. The idea of the simulation-cum-ROC approach is to consider only well-performing fit indices in the evaluation of model fit, with the best-performing fit index being the most decisive one.

In the following, we define those fit indices as well-performing that reach an AUC of at least .80 or higher, which aligns with earlier work (Padgett & Morgan, 2021). An AUC of .80 implies that 80% of the time, the fit index will correctly discriminate between correctly specified and misspecified models at different potential cutoffs (e.g., D’Agostino et al., 2013). Notably, the AUC threshold of .80 is not a universally valid one. We use it for illustrative purposes here. Depending on the specific application, a researcher may choose higher (stricter) or lower (more lenient) AUC thresholds—especially as type I and type II error rates of the corresponding cutoffs can exceed conventional levels of 5% or 10% at such an AUC threshold.^7^

Although we focus on well-performing fit indices with an AUC above a certain threshold (e.g., .80) to evaluate a model’s fit, inspecting the distributions of low-performing fit indices can also be informative. Strongly overlapping fit index distributions (i.e., AUC around .50) imply that a fit index cannot discern correctly specified from misspecified models. If few fit indices have strongly overlapping distributions, those particular fit indices might not be able to detect misfit in the scenario of interest. However, if several fit indices have strongly overlapping distributions, the misspecification of the analysis model relative to the *H*_*1*_ population model might not be strong enough to be detected in the scenario of interest. Similarly, the analysis model might be flexible enough to account for data from both *H*_*0*_ and *H*_*1*_ population models. Flexible (i.e., more complex) models are weaker than inflexible (i.e., less complex) ones, as flexible models fit a wide range of data (e.g., MacCallum, 2003). Thus, even strongly overlapping distributions (i.e., AUC around .50) may provide important insights.

Generally, it is important to bear in mind that different fit indices quantify different model, estimation, and data aspects (for an overview, see Schermelleh-Engel et al., 2003). For example, the ${\chi}^{2}$ test statistic (e.g., Bollen, 1989) quantifies the discrepancy between model-implied and sample-based variance-covariance matrix (with RMSEA being a transformation of it; Steiger, 1990). CFI indicates how well the model reproduces the sample-based variance-covariance matrix compared to a model where all items are uncorrelated (Bentler, 1990). SRMR quantifies the average residuals between model-implied and sample-based covariance matrices (Bentler, 1995). This is why fit indices perform differently well in different scenarios. Thus, the shape and overlap of distributions for each fit index help understand models (and accordingly misfit) further—as fit indices characterize models differently (see Browne et al., 2002; Lai & Green, 2016; Moshagen & Auerswald, 2018).

### Step 3: Generate tailored cutoffs

After identifying well-performing fit indices (e.g., AUC ≥ .80) and screening out the others, we can identify optimal cutoffs. To arrive at optimal cutoffs with the simulation-cum-ROC approach, we employ ROC analysis to select an optimal cutoff at the highest sum of sensitivity and specificity and, thus, the highest accuracy for each fit index. We interpret the type I error rate (i.e., 1 – specificity) and type II error rate (i.e., 1 – sensitivity) as equally problematic.^8^ At cutoffs with balanced type I and type II error rates, fit indices can best classify correctly specified models as correctly specified and misspecified models as misspecified.

We visualize the fit index distributions from correctly specified and misspecified models in a graph and provide cutoffs along with their accuracy, type I error rate, and type II error rate. Generally, the cutoff with the highest accuracy across fit indices belongs to the best-performing fit index (i.e., the one with the highest AUC).^9^ An essential advantage of the simulation-cum-ROC approach is that it returns the error rates associated with applying cutoffs. It draws researchers’ attention to how well cutoffs discriminate between correctly specified and misspecified models in the context of interest (quantified through type I and type II error rates).

Optimal cutoffs are not only identified in the simulation-cum-ROC approach but also in dynamic simulation approaches, though the strategies of the two approaches are different. The idea of the simulation-cum-ROC approach is to rank the fit indices by their performance. Optimal cutoffs are derived for all fit indices that meet an AUC threshold (commonly .80) and are, thus, considered well-performing. Common dynamic simulation approaches do not incorporate the feature to rank fit indices; optimal cutoffs are provided for all fit indices that meet conventional requirements of type I or type II error rates (commonly 5%/95% or 10%/90%, e.g., McNeish & Wolf, 2023a, b).

Thus, optimal cutoffs obtained via the simulation-cum-ROC approach do not need to meet certain requirements of type I or type II error rates and are typically derived at balanced error rates. However, those cutoffs might exceed conventional type I and type II error rates (i.e., 5%/95% and 10%/90%). Researchers then have the freedom to decide if they are willing to accept those so-obtained type I and type II error rates—and, accordingly, they have the freedom to use those so-obtained cutoffs.

Suppose researchers deem the type I and type II error rates to be too large. In that case, they need to impose stronger misspecification by redefining the *H*_*1*_ population model and, thus, adjust their assumptions about the level of misfit they want to reject. The derivation of tailored cutoffs needs to be redone. The initial and revised assumptions must be explicitly outlined and justified.

Thus, different from previous dynamic simulation approaches (e.g., McNeish & Wolf, 2023a, b), the simulation-cum-ROC approach allows for more researcher degrees of freedom but also forces the researcher to think about their choices regarding the hypotheses and models carefully, make them transparent, and provide a strong rationale for them.

### Output: Evaluate the fit of the analysis model to empirical data with tailored cutoffs

Having generated tailored cutoffs for well-performing fit indices with the simulation-cum-ROC approach, we can evaluate how well our analysis model (i.e., the one-factor model in our example) fits the empirical data by comparing the empirical values of the fit indices against the tailored cutoffs. In doing so, three scenarios may occur: (a) all fit indices point to good model fit, (b) all fit indices point to bad model fit, or (c) some fit indices point to good and some to bad model fit.

If all empirical values of fit indices pass the proposed tailored cutoffs, the analysis model has a good fit. Given the empirical data, *H*_*0*_ seems more plausible than *H*_*1*_. We can accept the analysis model. If all empirical values of fit indices fail the proposed tailored cutoffs, the analysis model has a poor fit. Given the empirical data, *H*_*1*_ seems more plausible than *H*_*0*_. We need to reject the analysis model.

There could be less straightforward empirical settings where the fit indices disagree (i.e., some pass, but others fail their respective cutoffs). In such cases, we can leverage the information from the ROC curve about the performance of fit indices uniquely provided by the simulation-cum-ROC approach. If there is a best-performing fit index and its empirical value suggests that the analysis model fits (i.e., it passes its tailored cutoff), *H*_*0*_ seems more plausible than *H*_*1*_. We accept the analysis model. If the best-performing fit index suggests that the analysis model does not fit, *H*_*1*_ seems more plausible than *H*_*0*_. We reject the analysis model. Thus, in those less-straightforward settings, we prioritize the best-performing fit index and its corresponding cutoff for our decision on model fit.

Rejecting the analysis model implies that the model is misspecified to the extent it was misspecified compared to the *H*_*1*_ population model—or even to a larger extent. Hence, rejecting the analysis model informs us about the severity of misspecification relative to the *H*_*1*_ population model. It does not inform us about the specific alternative model that has generated the data—this remains unknown as in all empirical settings.

If we reject the analysis model, we might want to modify it to find a better-fitting alternative. Modification indices help identify local misfit, though theory should also guide model modification (Fried, 2020). If theoretical and empirical indications lead to alterations of the analysis model, we need to test the modified model again. We must repeat the above procedure (Steps 1 to 3 of the simulation-cum-ROC approach) once we state a new *H*_*0*_ and *H*_*1*_.

## Application of the simulation-cum-ROC approach

In the following, we provide two examples that illustrate the simulation-cum-ROC approach. The aim of the first example is to illustrate the three steps to generate and apply tailored cutoffs. We chose a simple example without complications for the purpose of this illustration. All fit indices performed equally well in this example, which is not always guaranteed in real-life empirical applications.

The aim of the second example is to showcase the potential of the simulation-cum-ROC approach in ranking the performance of fit indices. In this example, the fit indices of interest differed in their performance; not all fit indices performed well enough to be useful for model evaluation.

We used publicly available secondary data for both examples (Nießen et al., 2018, 2020). We conducted all analyses with R (version 4.4.1; R Core Team, 2024). We used the R package lavaan to fit the models (version 0.6.19; Rosseel, 2012), simsem to simulate the data (version 0.5.16; Pornprasertmanit et al., 2021), pROC to plot the ROC curves (version 1.18.5; Robin et al., 2011), and cutpointr to obtain cutoffs for fit indices (version 1.1.2; Thiele & Hirschfeld, 2021). We documented all other packages used in the R code. Additional File 1 of the Supplementary Online Material includes the computational code.

We also programmed a Shiny app available under https://kg11.shinyapps.io/tailoredcutoffs/. Specifically, one needs to plug in their analysis model, population models, marginal skewness and excess kurtosis of the response distribution (used to obtain multivariate non-normal data with Vale and Maurelli’s method, 1983,10), estimator, sample size, number of simulation runs, fit indices one is interested in, and the AUC threshold. The Shiny app internally runs through Steps 1 to 3 of the simulation-cum ROC approach. It allows convenient downloading of the ROC curves from Step 2 and of the fit index distributions and tailored cutoffs from Step 3. Users need not execute any statistical program locally; the Shiny app does all the computational work to arrive at tailored cutoffs within the simulation-cum-ROC approach.

### Example 1: The Rosenberg Self-Esteem Scale

We chose the Rosenberg Self-Esteem Scale as a first example for generating tailored cutoffs via the simulation-cum-ROC approach (Rosenberg, 1965). The Rosenberg Self-Esteem Scale measures global self-esteem with ten items (five referring to positive feelings and five to negative ones) rated on a four-point Likert scale. Initially thought to measure a single factor, later studies found evidence for a two-factor structure (or even more complex structures; see Supple et al., 2013, for an overview). In this example, we focused only on one of the two factors, the one for negative feelings, and evaluated its unidimensionality. We used publicly available data (*N* = 468; Nießen et al., 2020) that contains the Rosenberg Self-Esteem Scale applied to a quota sample of adults aged 18 to 69 from the United Kingdom.

**Fig. 3:**
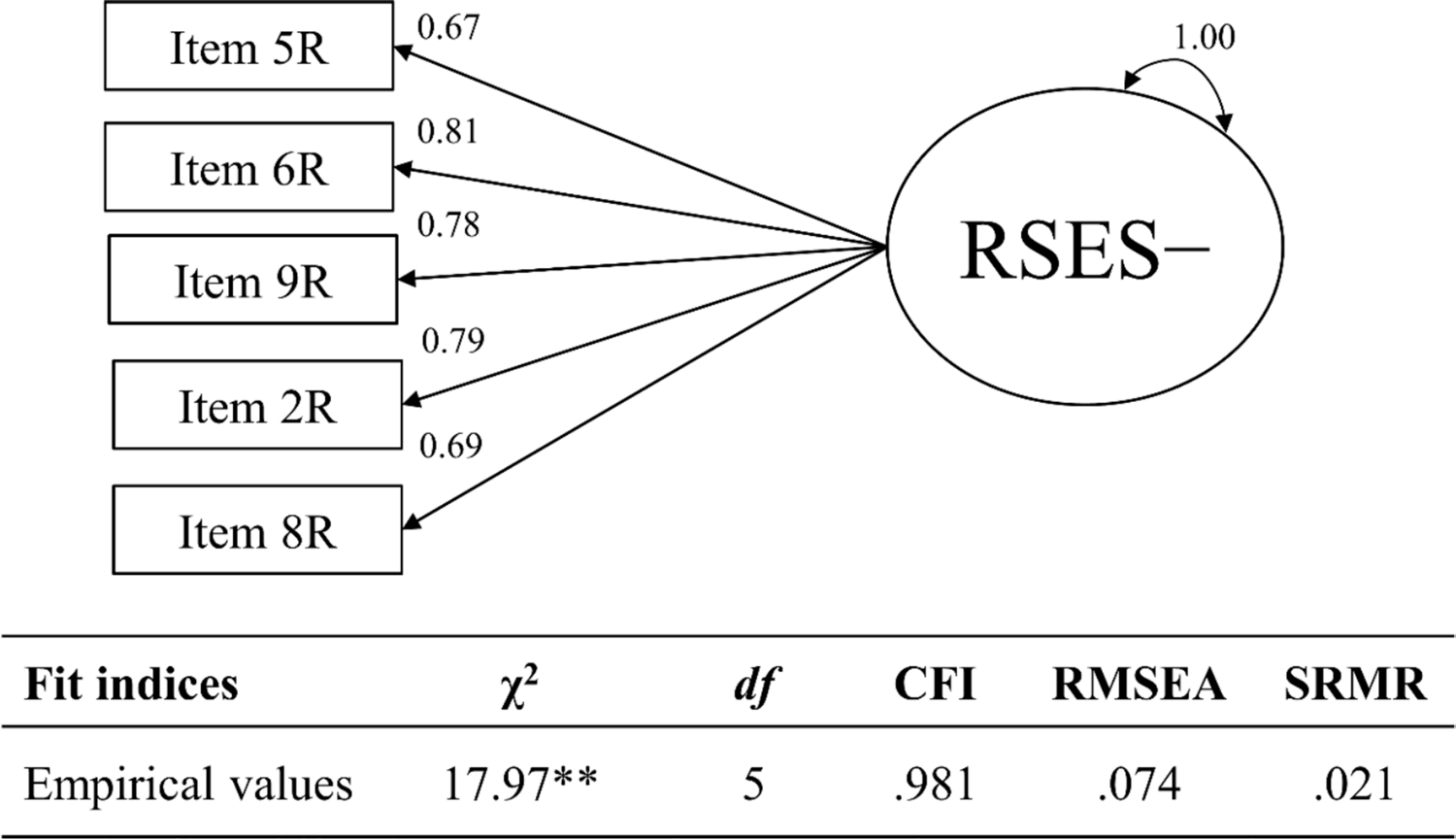
Empirical one-factor Rosenberg Self-Esteem Scale model (negative feelings). *Note.* Unstandardized coefficients. RSES = Rosenberg Self-Esteem Scale. We recoded the items so that higher values imply higher self-esteem. We omitted the residual variances and the mean structure for clarity. *N* = 468. ** *p* < .01

***Input: Fit analysis model to empirical data.*** We fit the one-factor model to the empirical data using MLR. Figure 3 depicts the analysis model and the empirical fit index values. We evaluated whether empirical evidence favors *H*_*0*_ or *H*_*1*_ for the one-factor model using tailored cutoffs. We would accept the one-factor model if empirical evidence favored *H*_*0,*_ stating that a population model identical (or nearly identical) to the one-factor model had generated the data; the one-factor model captured all relevant features of the population model. We would reject the one-factor model if empirical evidence favored *H*_*1,*_ stating that a population model different from the one-factor model had generated the data; a one-factor model failed to capture relevant features of the population model to an intolerable degree.

#### Step 1: Simulate data and fit analysis model to simulated data

After fitting the one-factor model to empirical data, we defined *H*_*0*_ and* H*_*1*_ for the Monte Carlo simulation. The one-factor model served as an analysis model in the simulation. The structure and parameter estimates of the one-factor model fit to empirical data served as the *H*_*0*_ population model. Relative to the one-factor population model, the analysis model was correctly specified.

**Fig. 4:**
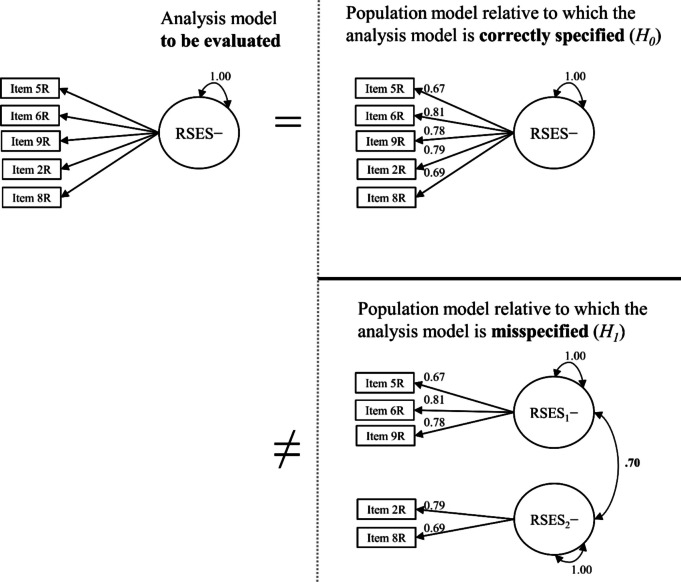
Proposed analysis and population models of the Rosenberg Self-Esteem Scale (negative feelings). *Note.* Unstandardized coefficients. RSES = Rosenberg Self-Esteem Scale. We recoded the items so that higher values imply higher self-esteem. We omitted the residual variances and the mean structure for clarity

Next, we must define a theoretically justifiable *H*_*1*_ population model. A good candidate for an *H*_*1*_ population model could be a two-factor rather than a one-factor model, as the question of dimensionality is at the core of model testing (Brown, 2015). We set the factor correlation between two factors (which equals 1 for the one-factor model) to .70, inducing a misspecification of *r* = .30, a correlation considered medium (Cohen, 1992). Thus, we chose an *H*_*1*_ population model identical to the *H*_*0*_ population model (and, thus, the analysis model) in the parameter estimates (i.e., loadings and residual variances) but split into two factors correlating at .70 (with one factor containing the items explicitly referring to feelings). Relative to the two-factor population model, the analysis model was underspecified (i.e., misspecified). Figure 4 shows the population and analysis models.

We simulated data from the *H*_*0*_ and *H*_*1*_ population models, fit the one-factor analysis model to that data, and recorded the fit index values. The Monte Carlo simulation closely resembled the empirical setting regarding the sample size (i.e., *N* = 468), the estimator of choice (i.e., MLR), and the multivariate response distribution. We simulated data 500 times from each population model.

#### Step 2: Evaluate the performance of fit indices

**Fig. 5:**
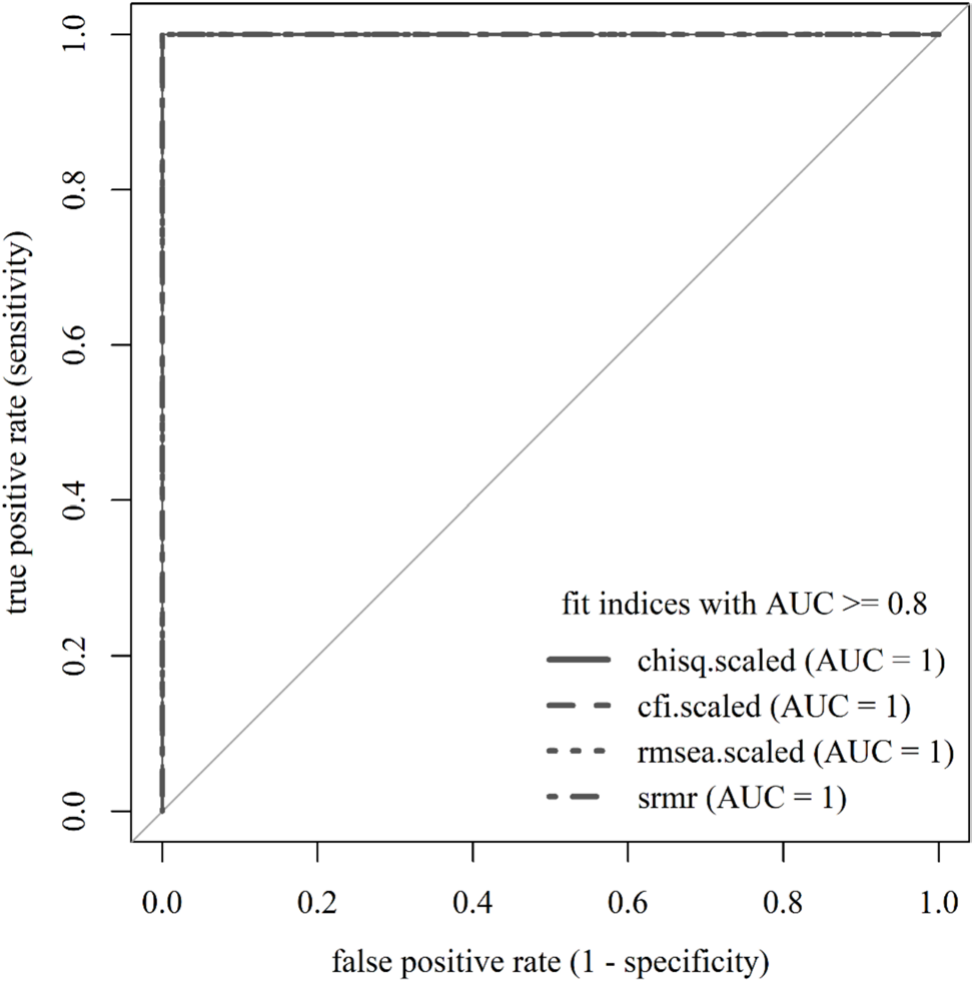
ROC curves for fit indices with AUC ≥ .80 of the Rosenberg Self-Esteem Scale model (negative feelings). *Note.* Chisq.scaled is a ${\chi}^{2}$ test statistic asymptotically equivalent to the robust Yuan–Bentler test statistic (Yuan & Bentler, 2000a) to account for non-normality. Cfi.scaled is the CFI version and rmsea.scaled is the RMSEA version calculated with this test statistic

After simulating the data, we evaluated the performance of fit indices as quantified through the AUC. We only considered fit indices with an AUC of .80 or higher (Padgett & Morgan, 2021) and disregarded all others. Figure 5 displays the ROC curves of the fit indices (in different line shapes). In Fig. 5, the different lines representing the different ROC curves completely overlap for all fit indices. All fit indices had an AUC equal to or higher than .80, namely an AUC of 1. Thus, the ROC curves and AUCs were the same for *all* fit indices, which implies that all fit indices discriminated equally well between correctly specified and misspecified models. This is certainly not always the case, as shown in the second example.

#### Step 3: Generate tailored cutoffs

**Fig. 6:**
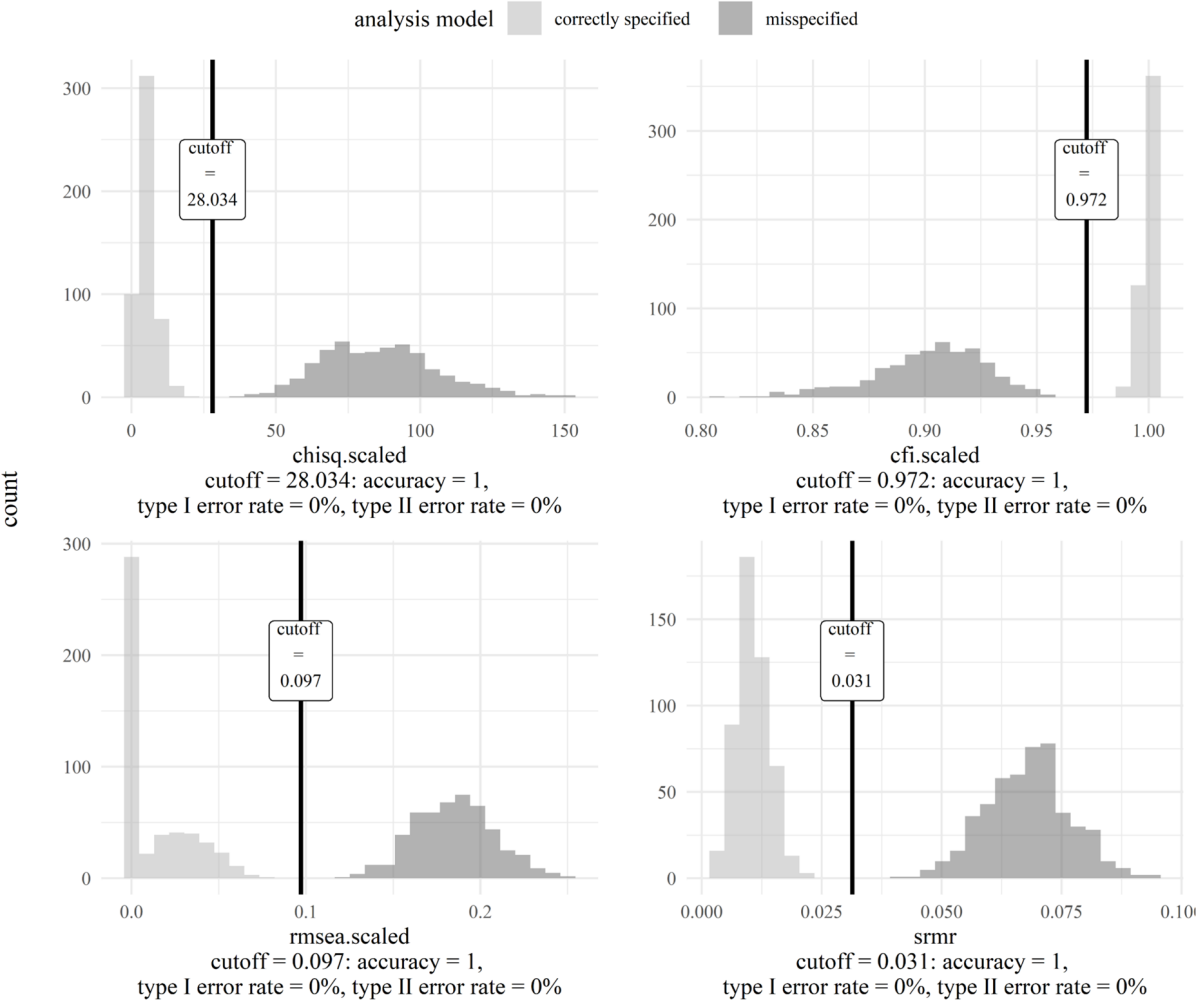
Cutoffs for fit indices with AUC ≥ .80 of the Rosenberg Self-Esteem Scale model (negative feelings). *Note.* Chisq.scaled is a ${\chi}^{2}$ test statistic asymptotically equivalent to the robust Yuan–Bentler test statistic (Yuan & Bentler, 2000a) to account for non-normality. Cfi.scaled is the CFI version and rmsea.scaled is the RMSEA version calculated with this test statistic. The distribution colored in lighter gray originates from correctly specified models. The distribution colored in darker gray originates from misspecified models. Overlapping (parts of) distributions are colored in an even darker gray than the distribution from misspecified models. The vertical dash corresponds to the cutoff for each fit index (at the highest sum of sensitivity and specificity – 1)

In Step 3, we generated cutoffs for well-performing fit indices. All fit indices performed equally well (as quantified through the AUC). Thus, we generated tailored cutoffs for all fit indices. Figure 6 depicts the fit index distributions for the simulated data. The distribution colored in lighter gray is the one for fit index values from correctly specified models. The distribution colored in darker gray is the one for fit index values from misspecified models. The vertical dash corresponds to the cutoff (maximizing the sum of sensitivity and specificity $-$ 1).^11^ The cutoffs were the following: ${\chi}^{2}$(5) ≤ 28.03, CFI ≥ .972, RMSEA ≤ .097, SRMR ≤ .031. All cutoffs across fit indices had an accuracy of 1. Type I and type II error rates were zero for all cutoffs. Thus, all cutoffs perfectly discriminated between correctly specified and misspecified models in this scenario. Again, perfectly discriminating cutoffs do not reflect the usual case, as shown in the second example.

#### Output: Evaluate the fit of the analysis model to empirical data with tailored cutoffs

Judged against the tailored cutoffs, we accepted the one-factor model for the negative feelings of the Rosenberg Self-Esteem Scale fit to empirical data. All of the empirical fit index values for the one-factor model (${\chi}^{2}$(5) = 17.97, *p* < .01; CFI = .981; RMSEA = .074; SRMR = .021) passed the tailored cutoffs (i.e., ${\chi}^{2}$(5) ≤ 28.03; CFI ≥ .972; RMSEA ≤ .097; SRMR ≤ .031). Given the empirical data, *H*_*0*_ seemed more plausible, stating that the one-factor model generated the data.

Whereas according to fixed cutoffs of CFI around .950 and SRMR around .080 (but not RMSEA around .060; Hu & Bentler, 1999), the one-factor would also fit and be accepted, we were more confident that the tailored cutoffs correctly classified the one-factor model as correctly specified. Those fixed cutoffs were generated from three-factor models with 15 items in total (Hu & Bentler, 1999)—largely different from the empirical setting at hand. The tailored cutoffs applied here, in turn, were explicitly targeted at our one-factor model with five items (and all the other characteristics of the empirical setting at hand). Additionally, we knew that all fit indices performed equally well and are, thus, equally decisive for model evaluation. This question would be left unanswered with fixed cutoffs for fit indices as well as other approaches to tailored cutoffs.

### Example 2: The social desirability-gamma short scale

To illustrate the potential of the simulation-cum-ROC approach, we took the Social Desirability-Gamma Short Scale (Kemper et al., 2014; Nießen et al., 2019) as a second example. Paulhus’s (2002) theoretical model of socially desirable responding was the basis for this scale. Socially desirable responding refers to deliberate attempts to present oneself in a favorable light (e.g., as a nice person or good citizen). The Social Desirability-Gamma Short Scale measures the two aspects of the Gamma factor of socially desirable responding: exaggerating one’s positive qualities (PQ+) and minimizing one’s negative qualities (NQ−) with three items each. Respondents rate these items on a five-point Likert scale. Publicly available data (*N* = 474; Nießen et al., 2018) contains the German version of the scale applied to a quota sample of adults aged 18 to 69 years in Germany.

#### Input: Fit analysis model to empirical data

**Fig. 7:**
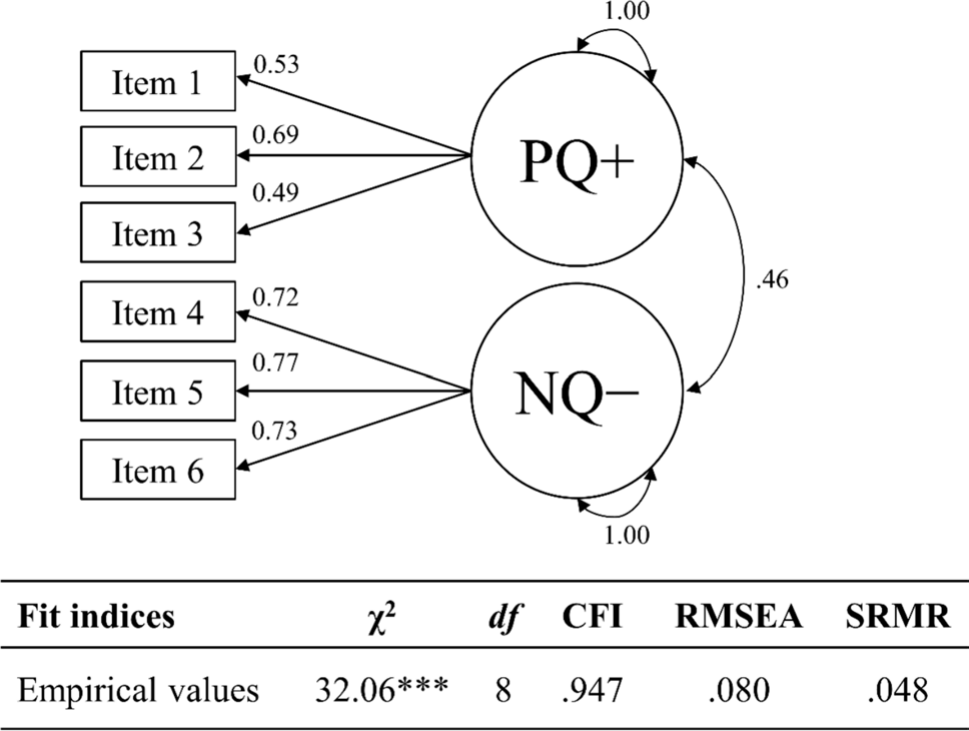
Empirical two-factor Social Desirability-Gamma Short Scale model. *Note.* Unstandardized coefficients. PQ+ = exaggerating positive qualities; NQ− = minimizing negative qualities. We recoded NQ− so that higher values imply more socially desirable responses. We omitted the residual variances and the mean structure for clarity. *N* = 474. *** *p* < .001

We fit the two-factor model of the Social Desirability-Gamma Short Scale to the empirical data using MLR (following Nießen et al., 2019). Figure 7 depicts the two-factor model and its empirical values of fit indices. We evaluated whether empirical evidence favors *H*_*0*_ or *H*_*1*_ for the two-factor model using tailored cutoffs. We would accept the two-factor model if empirical evidence favored *H*_*0,*_ stating that a population model identical (or nearly identical) to the two-factor model had generated the data; the two-factor model captured all relevant features of the population model. We would reject the two-factor model if empirical evidence favored *H*_*1,*_ stating that a population model different from the two-factor model had generated the data; a two-factor model failed to capture relevant features of the population model to an intolerable degree.

#### Step 1: Simulate data and fit analysis model to simulated data

After fitting the two-factor model to empirical data, we defined *H*_*0*_ and* H*_*1*_ for the Monte Carlo simulation. The two-factor model served as an analysis model in the simulation. The structure and parameter estimates of the two-factor model fit to empirical data served as the *H*_*0*_ population model. Relative to the *H*_*0*_ population model, the analysis model was correctly specified.

**Fig. 8:**
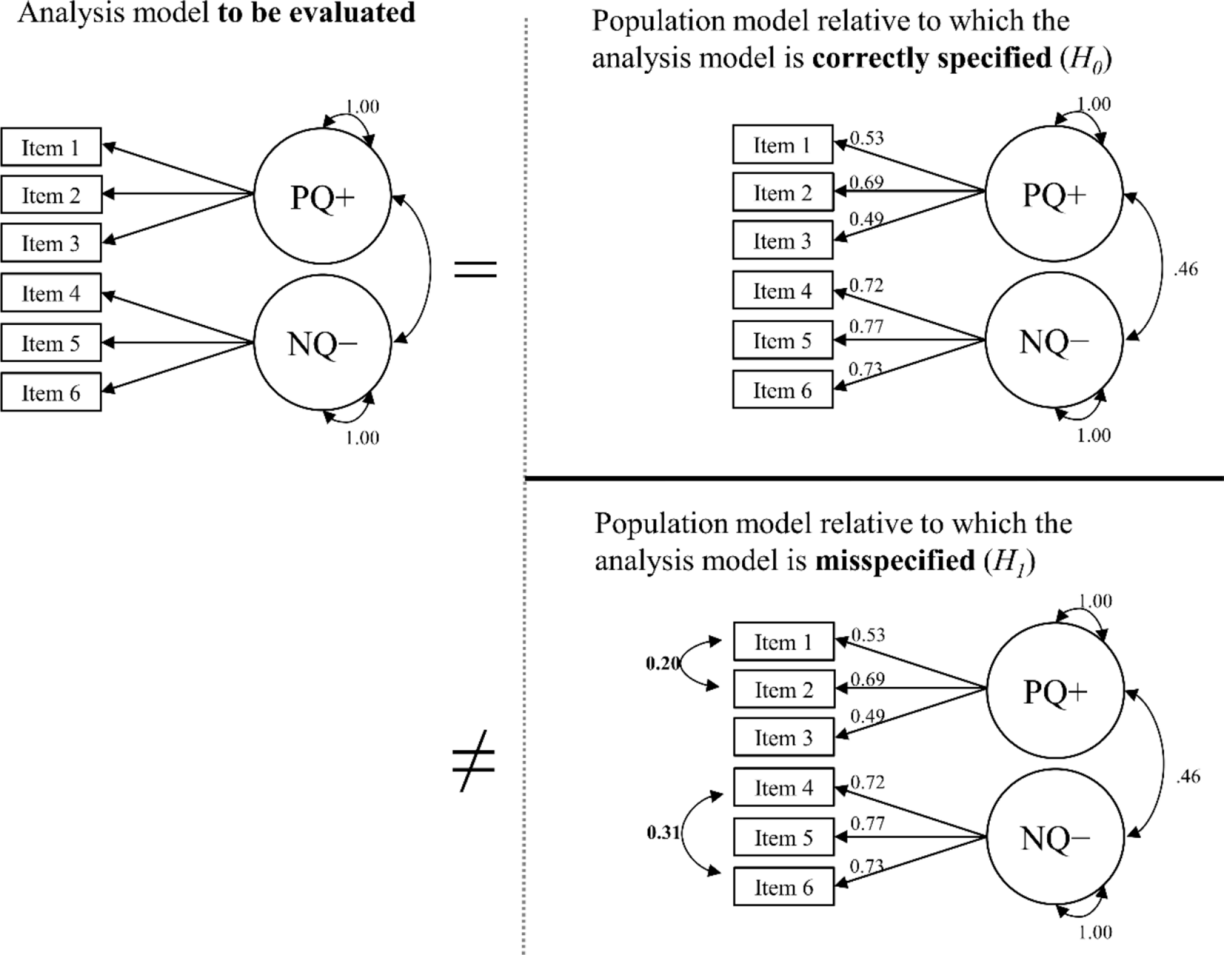
Proposed analysis and population models of the Social Desirability-Gamma Short Scale. *Note.* Unstandardized coefficients. PQ+ = exaggerating positive qualities; NQ− = minimizing negative qualities. We recoded NQ− so that higher values imply more socially desirable responses. We omitted the residual variances and the mean structure for clarity

Next, we must define a theoretically justifiable *H*_*1*_ population model. A good candidate for an *H*_*1*_ population model could be a two-factor model that contains additional residual covariances to capture shared wording effects. The question of whether additional residual covariances are needed to fully account for the covariances among items is one with which applied researchers frequently grapple (e.g., Bluemke et al., 2016; Podsakoff et al., 2003). Correlations of *r* = .50 have been considered large (Cohen, 1992). Two unmodeled residual correlations have been considered moderate misspecification for six-item models (McNeish & Wolf, 2023a). We chose an *H*_*1*_ population model that was identical to the *H*_*0*_ population model (and, thus, the analysis model) in the latent-variable part but comprised two residual correlations of *r* = .50 each. We modeled one residual correlation between the first two items of the PQ+ factor (resulting in a residual covariance of 0.20), both asking for emotional control. We modeled another residual correlation between the first and third items of the NQ− factor (resulting in a residual covariance of 0.31), both referring to behavior in social interactions. Relative to this *H*_*1*_ population model, the analysis model was underspecified (i.e., misspecified). Figure 8 shows the population and analysis models for examining *H*_*0*_ and *H*_*1*_.

We simulated data from the population models, fit the analysis model to each simulated data, and recorded the fit indices. Essential features for the simulation mimicked the empirical setting in terms of the sample size (i.e., *N* = 474), estimator (i.e., MLR), and the multivariate response distribution. We simulated data 500 times from each population model.

#### Step 2: Evaluate the performance of fit indices

**Fig. 9:**
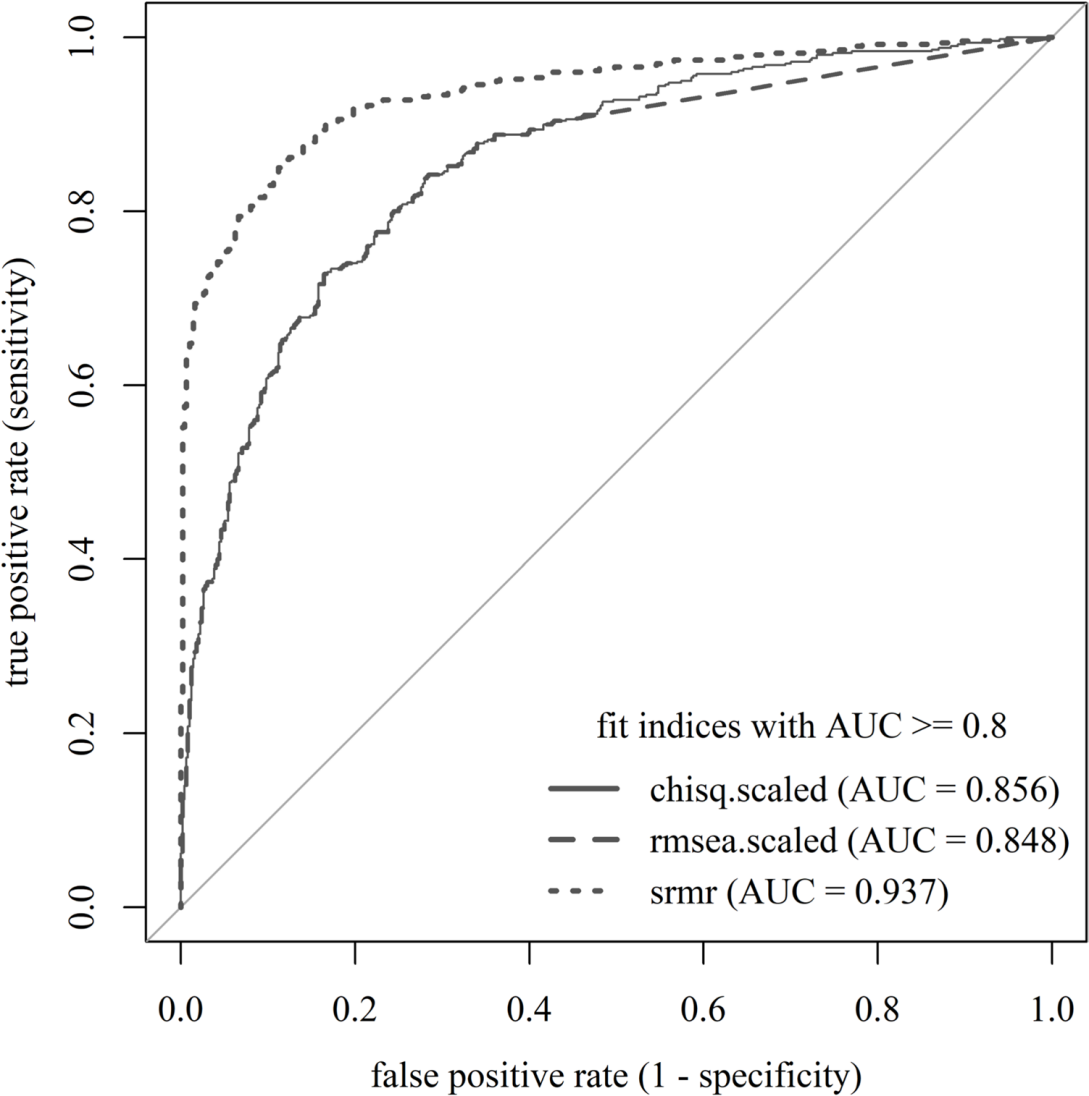
ROC curves for fit indices with AUC ≥ .80 of the Social Desirability-Gamma Short Scale model. *Note.* Chisq.scaled is a ${\chi}^{2}$ test statistic asymptotically equivalent to the robust Yuan–Bentler test statistic (Yuan & Bentler, 2000a) to account for non-normality. Rmsea.scaled is the RMSEA version calculated with this test statistic

Unlike the previous example, not all fit indices passed the AUC ≥ .80 benchmark, and the AUCs were generally lower (i.e., below 1). Figure 9 visualizes the ROC curves of three fit indices with an AUC of .80 or higher: ${\chi}^{2}$, RMSEA, and SRMR. We disregarded CFI because, with an AUC below .80, it did not perform adequately in this scenario. Among the three well-performing fit indices with AUC ≥ .80 (i.e., ${\chi}^{2}$, RMSEA, and SRMR but not CFI), SRMR had the highest AUC (= .94) and was, thus, the best-performing fit index in our scenario.

#### Step 3: Generate tailored cutoffs

**Fig. 10:**
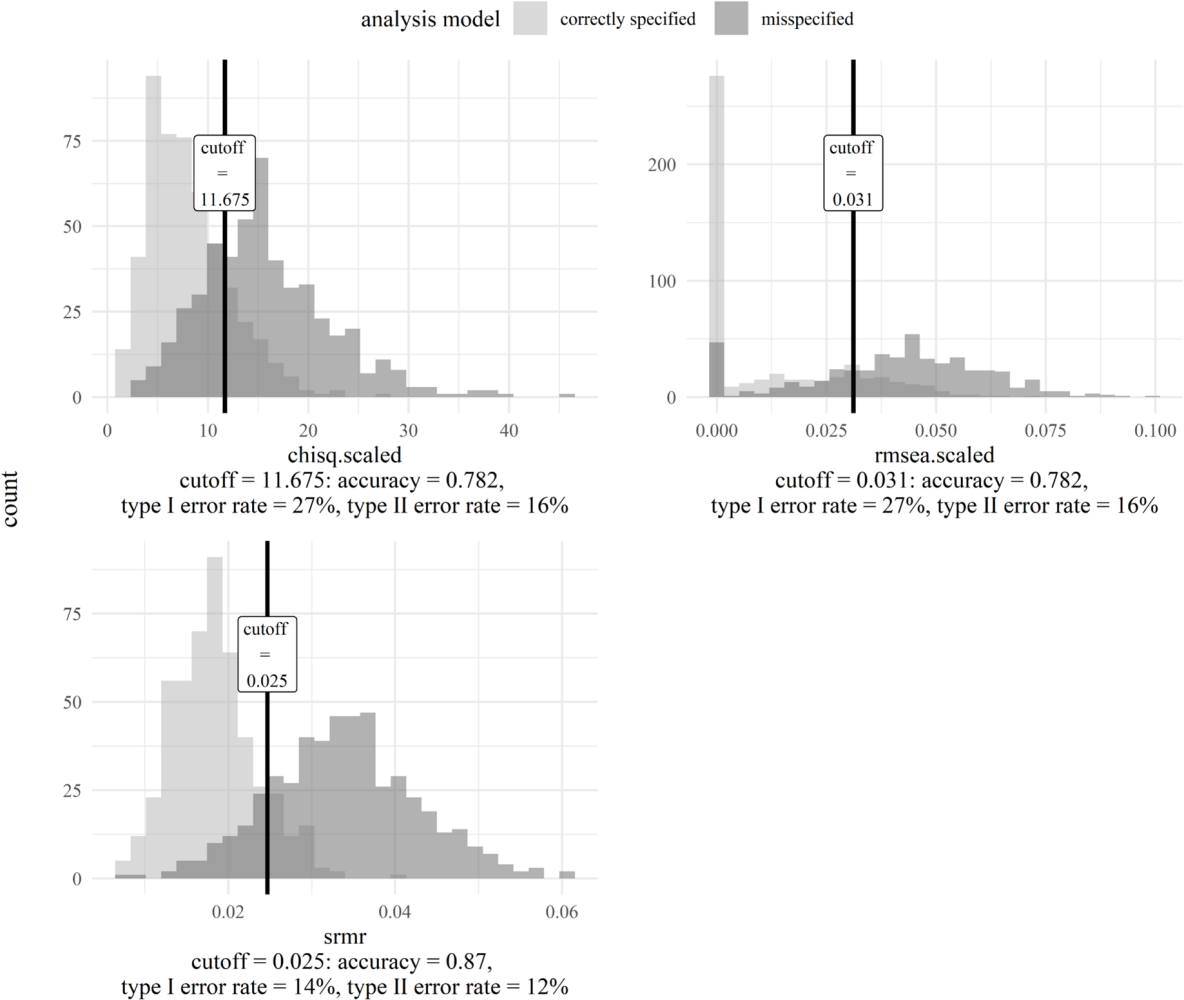
Cutoffs for fit indices with AUC ≥ .80 of the Social Desirability-Gamma Short Scale model. *Note.* Chisq.scaled is a ${\chi}^{2}$ test statistic asymptotically equivalent to the robust Yuan–Bentler test statistic (Yuan & Bentler, 2000a) to account for non-normality. Rmsea.scaled is the RMSEA version calculated with this test statistic. The distribution colored in lighter gray originates from correctly specified models. The distribution colored in darker gray originates from misspecified models. Overlapping (parts of) distributions are colored in an even darker gray than the distribution from misspecified models. The vertical dash corresponds to the cutoff for each fit index (at the highest sum of sensitivity and specificity – 1)

We generated cutoffs only for the three well-performing fit indices in the following. The cutoff for ${\chi}^{2}$ was 11.68, RMSEA .031, and SRMR .025 (Fig. 10). In line with the AUC, the cutoff for SRMR had the highest accuracy (= .87) as well as the lowest type I error rate (= 14%) and type II error rate (= 12%). It better categorized correctly specified models as correctly specified and misspecified models as misspecified than cutoffs for other fit indices. Thus, the SRMR, with its corresponding cutoff, had the best ability to demarcate between correctly specified and misspecified models in the scenario of interest. Further, it implies that the greatest difference between correctly specified and misspecified models in the specific scenario was due to average standardized residuals.

The reader may have noted that these cutoffs’ type I and type II error rates are above conventional levels of 5% or 10%. If we deem the error rates too high, we can redefine the *H*_*1*_ population model. To redefine the *H*_*1*_ population model, we need to repeat Steps 1 through 3 of the simulation-cum-ROC approach: In Step 1, we need to define a new *H*_*1*_ population model, from which the analysis model is “further” away than the initial *H*_*1*_ population model. For instance, the new *H*_*1*_ population model contains more or higher non-zero parameter values than the initial *H*_*1*_ population model, which the analysis model wrongly fixes to zero.

Alternatively, we can use the cutoffs while accepting their given error rates. Here, we deemed the error rates acceptable (especially the ones of SRMR) because we explicitly wanted to retain the definitions of population models as outlined and justified in this example. Imposing stronger misspecification through redefining the *H*_*1*_ population model would lead to more lenient cutoffs than the current ones. This would imply that those cutoffs might lead to accepting an empirical model that contains misfit of a size that we initially deemed unacceptable (i.e., through the initial definition of the *H*_*1*_ population model relative to which the analysis model is misspecified).

#### Output: Evaluate the fit of the model to empirical data with tailored cutoffs

When comparing the empirical fit index values to the cutoffs tailored to the setting of interest, we rejected the two-factor model of the Social Desirability-Gamma Short Scale. The empirical values of fit indices (${\chi}^{2}$(8) = 32.06, *p* < .001; CFI = .947; RMSEA = .080; SRMR = .048) clearly failed all tailored cutoffs (${\chi}^{2}$(8) ≤ 11.68; CFI = should not be considered; RMSEA ≤ .031; SRMR ≤ .025). Thus, *H*_*1*_ seemed more plausible than *H*_*0*_, concluding that a model different from a two-factor one is likely to have generated the data.

Notably, fixed cutoffs of CFI around .950 and SRMR around .080 (but not RMSEA around .060; Hu & Bentler, 1999) were far off the tailored cutoffs and would wrongly lead to accepting the two-factor model. This underscores that fixed cutoffs would not have provided valid assessments of model fit in settings markedly different from the simulation scenarios they originated from (i.e., three-factor models with 15 items). Additionally, we knew that the SRMR was most decisive for decisions about model fit (if fit indices would disagree about model acceptance or rejection)—something that would remain unknown with fixed cutoffs for fit indices and other approaches to tailored cutoffs.

As we rejected the two-factor model, we must modify the model and test the modified model again. A modified model can be considered a new empirical setting, so testing that modified model requires a new set of tailored cutoffs. We demonstrated how to employ the simulation-cum-ROC approach to test a modified Social Desirability-Gamma Short Scale model for interested readers in Additional File 2 of the Supplementary Online Material. We made use of the different performance of fit indices in that example as their decisions on model fit disagreed.

## Discussion

Fixed cutoffs for fit indices are far more problematic than many researchers realize (e.g., Groskurth et al., 2024; Marsh et al., 2004; Lai & Green, 2016). Fixed cutoffs have low external validity and do not generalize well to settings not covered in simulation studies from which these cutoffs originate. This is because fit indices are susceptible to various influences other than model misspecifications they should detect (for an overview, see Groskurth et al., 2024; Niemand & Mai, 2018; McNeish & Wolf, 2023a, b; Pornprasertmanit, 2014). Cutoffs tailored to the setting of interest are generally more appropriate than fixed cutoffs whenever the setting falls outside the limited range of simulation scenarios from which these cutoffs were derived (such as those by Hu and Bentler, 1999). Therefore, methodologists are increasingly urging that fixed cutoffs should be abandoned and replaced by tailored (or “dynamic”) cutoffs (e.g., Markland, 2007; Marsh et al., 2004; McNeish & Wolf, 2023a; Niemand & Mai, 2018; Nye & Drasgow, 2011).

The current article reviewed four principal approaches to generating tailored cutoffs. This is the first article to comprehensively review and synthesize the approaches to tailored cutoffs. While we have outlined their strengths and limitations on a conceptual level, future research may additionally want to compare their performance statistically. For example, simulation studies comparing type I and type II error rates of cutoffs generated from the various approaches in different contexts have yet to be conducted.

We then introduced a novel approach, the simulation-cum-ROC approach, that augments the dynamic simulation approach to tailored cutoffs that has gained traction in recent literature (e.g., McNeish & Wolf, 2023a, b; Millsap, 2013; Niemand & Mai, 2018). By applying ROC analysis to distributions of fit indices from a Monte Carlo simulation, the simulation-cum-ROC approach provides a highly informative way to evaluate model fit. Like several other approaches outlined in our review, the simulation-cum-ROC approach generates (1) tailored cutoffs at certain type I and type II error rates (i.e., balanced ones for the simulation-cum-ROC approach) for several fit indices across different settings. However, it conceptually advances previous approaches by (2) ranking the performance of fit indices (i.e., their ability to discriminate between correctly specified and misspecified models) for the specific setting of interest. Thus, the unique strength of the simulation-cum-ROC approach is that it provides guidance on which fit index to rely on (or at least assign the greatest weight) when evaluating model fit in the specific setting of interest.

To illustrate how our proposed simulation-cum-ROC approach works, we tested models of the Rosenberg Self-Esteem Scale and the Social Desirability-Gamma Short Scale. We wish to emphasize that we intended these examples as proof of principle. In presenting these examples, we made several choices on the selection of fit indices, the definition of population models, and the relative importance of type I and type II error rates in generating tailored cutoffs. Researchers can modify most of these choices when applying the proposed simulation-cum-ROC approach to other empirical problems. We highlight some of these choices to underscore our approach’s generality and identify areas in which future research may progress.

To begin with, researchers may consider additional variants of fit indices or different fit indices altogether. In our examples, we focused on the three widely used fit indices, CFI, RMSEA, and SRMR (Jackson et al., 2009), to keep these examples simple. Additionally, as is routine in applied research, we considered ${\chi}^{2}$ in much the same way (and not as a strict formal test; see Jöreskog & Sörbom, 1993).^12^ We relied on a ${\chi}^{2}$ test statistic approximately equivalent to the Yuan–Bentler one (Yuan & Bentler, 2000a; called chisq.scaled in lavaan, see also Savalei & Rosseel, 2022). Following standard practice (e.g., Muthén & Muthén, 1998–2017), we relied on the CFI and RMSEA versions calculated with this ${\chi}^{2}$ test statistic (called cfi.scaled and rmsea.scaled in lavaan). The standard formulations of fit indices (and test statistics) are not without criticism. Several authors (Brosseau-Liard et al., 2012; Brosseau-Liard & Savalei, 2014; Gomer et al., 2019; Yuan & Marshall, 2004; Yuan, 2005; Zhang, 2008) have pointed out problems and suggested improved formulations. Therefore, researchers may prefer not to go with the conventional fit indices we used in the examples. Notably, the simulation-cum-ROC approach can be generalized to include any other fit index, including variants of the canonical fit indices (e.g., Yuan, 2005) but also other, less widely used fit indices (e.g., McDonald’s measure of centrality, McDonald, 1989, or the adjusted goodness of fit index, Jöreskog & Sörbom, 1986).

Moreover, in our examples of the simulation-cum-ROC approach, we chose an AUC value of .80 as a threshold. Researchers may choose higher AUC thresholds for lower type I and type II error rates. Moreover, we selected a cutoff as the optimal one that had the highest sum of sensitivity + specificity – 1 (i.e., the Youden index balancing type I and type II error rates). Alternatively, researchers might maximize sensitivity given a minimal specificity value to obtain optimal cutoffs (or vice versa).

We provided R code in Additional File 1 of the Supplementary Online Material and programmed a Shiny app available under https://kg11.shinyapps.io/tailoredcutoffs/ to ease the application of the simulation-cum-ROC approach. Executing the simulation-cum-ROC approach for our examples took two to three minutes on a standard computer using R (single-threaded).

It is essential to realize that tailored cutoffs derived from the simulation-cum-ROC approach are the most accurate decision thresholds for the setting from which they originate. That said, one should not make the same mistake as with traditional cutoffs and generalize tailored cutoffs to any different combination of model, estimation, and data characteristics. Different combinations affect the performance of fit indices and their cutoffs in unexpected and non-traceable ways (for an overview, see Niemand & Mai, 2018; Pornprasertmanit, 2014), and erroneous conclusions may result. We instead underline that no general cutoff or general statement on the performance of those commonly used fit indices exists (see also, e.g., Marsh et al., 2004; Nye & Drasgow, 2011; McNeish & Wolf, 2023a).

### Advanced definitions of population models

A challenge in applying the simulation-cum-ROC approach—one that it shares with similar dynamic simulation approaches (e.g., Pornprasertmanit, 2014)—concerns the definition of the *H*_*0*_ and *H*_*1*_ population models (cf. McNeish & Wolf, 2023a, b, who already predefined *H*_*0*_ and *H*_*1*_ population models). More advanced definitions of population models can be easily integrated into the simulation-cum-ROC approach. For example, one could define an *H*_*0*_ population model relative to which an analysis model is negligibly underspecified (i.e., misspecified) to test for approximate fit, as suggested by Millsap (2007, 2013) and Pornprasertmanit (2014). We indeed believe that alternative definitions of population models can be fruitful, so we briefly review possible extensions of our approach (and similar approaches) that have been proposed in prior work. We further identify areas in which future work on generating tailored cutoffs could make further progress.

### Approximate fit

In our examples illustrating the simulation-cum-ROC approach, the analysis models were always identical to the *H*_*0*_ population models. We generated cutoffs based on an analysis model that exactly fits the data generated by (i.e., simulated from) that *H*_*0*_ population model. Only sampling fluctuations influenced the resulting fit index distributions and, accordingly, the cutoffs (Cudeck & Henly, 1991; MacCallum, 2003; MacCallum & Tucker, 1991). Testing the assumption of exact fit has guided model evaluation for years; the entire distributional assumptions of the ${\chi}^{2}$ test statistic rely on the test of exact fit (e.g., Bollen, 1989). Testing exact fit is legitimate if the aim is to find a model that perfectly describes the specific population. This model should perfectly reproduce all major and minor common factors in the specific data.

In empirical applications, researchers commonly want to find models (more precisely, specific features, e.g., broad factors in models) that do not solely reproduce a specific population but are generalizable to different populations (a broad array of, e.g., demographic populations; Cudeck & Henly, 1991; Millsap, 2007; see also Wu & Browne, 2015). In other words, researchers do not want to find an overfitting model. Toward that end, it can be advantageous to consider not only sampling fluctuations but also model error when generating cutoffs (Cudeck & Henly, 1991; MacCallum, 2003; MacCallum & Tucker, 1991). In this context, model error means choosing an *H*_*0*_ population model relative to which the analysis model already contains minor misspecification, such as small unmodeled residual correlations (e.g., Millsap, 2007, 2013). The analysis model is underspecified (i.e., misspecified) to a certain degree relative to the *H*_*0*_ population model. Researchers still consider the analysis model correctly specified, barring trivial misspecification they deem acceptable. It is within their realistic expectations of how well a model can capture the complexities of a real population while still being plausible in others (for an overview and more in-depth discussion, see MacCallum, 2003; see Wu & Browne, 2015, for the related concept of adventitious error that defines the differences between the sampled and theoretically hypothesized population). Including model error (in addition to sampling fluctuations) in the derivation of cutoffs is known as testing approximate fit and has already been implemented in several approaches (e.g., Kim & Millsap, 2014; McNeish & Wolf, 2023a; Millsap, 2013; Yuan & Hayashi, 2003; Yuan et al., 2004, 2007).

We opted against testing approximate fit in our two examples for didactic reasons (i.e., to keep the exposition simple). However, for interested readers, we included an additional example that illustrates how to select the *H*_*0*_ population model such that one tests approximate (instead of exact) fit in Additional File 2 of the Supplementary Online Material. As the example demonstrates, testing approximate fit via the simulation-cum-ROC approach works much the same way as testing exact fit and poses no additional hurdle.

### Multiple population models

So far, we have always defined a single *H*_*0*_* / H*_*1*_ population model to test the fit of an analysis model of interest. As defined by Pornprasertmanit (2014; see also Pornprasertmanit et al., 2013), we followed the *fixed method* (see also Millsap, 2013)*.* By following the fixed method (i.e., defining only a single *H*_*0*_* / H*_*1*_ population model), we take only one form of misspecification (e.g., omitted residual correlation of *r* = .50) out of all possible misspecifications in the space of conceivable models into account.

To ensure decisions about accepting or rejecting a model are generalizable to other forms of misspecification, we could, for example, repeatedly follow the fixed method and conduct so-called robustness checks. To conduct robustness checks, we define different forms of misspecification and derive new cutoffs for each of them. The degree of misspecification should roughly stay the same to compare the cutoffs’ robustness across different forms of misspecification. These robustness checks investigate whether we will make the same decision about accepting or rejecting a model with different forms of misspecification. We included an example of a robustness check for the Social Desirability-Gamma Short Scale example in Additional File 2 of the Supplementary Online Material. In the Guidelines on Forms and Degrees of Misspecification section, we provide some guidance on defining forms and quantifying degrees of misspecification.

Pornprasertmanit (2014) proposed new methods that directly take a wide variety of misspecification forms into account (e.g., omitted residual correlations; omitted cross-loadings) when deriving a set of cutoffs (ideally at the same degree of misspecification). Like the robustness checks for the fixed method, the new methods apply to both *H*_*0*_ population models (to test approximate fit) and *H*_*1*_ population models. Recall that the *H*_*0*_ population model implies trivial, acceptable misspecification (to test approximate fit), and the* H*_*1*_ population model implies severe, unacceptable misspecification of the analysis model relative to that population model.

In the *random method*, one defines several *H*_*0*_ / *H*_*1*_ population models relative to a misspecified analysis model. The analysis model is trivially misspecified to *H*_*0*_ population models and severely misspecified to *H*_*1*_ population models. The algorithm randomly picks a new *H*_*0*_ / *H*_*1*_ population model from the several possible *H*_*0*_ / *H*_*1*_ population models (defined initially) each time it starts simulating data. This approach considers multiple *H*_*0*_ / *H*_*1*_ population models relative to a misspecified  analysis model. The population models are the same for different fit indices but differ across simulation runs.

In the *maximal method* (for defining *H*_*0*_ population models) or the *minimal method* (for defining *H*_*1*_ population models), one again defines several *H*_*0*_ / *H*_*1*_ population models relative to a misspecified analysis model. Then, one draws data from all those population models and fits the analysis model to the data. When selecting an *H*_*0*_ population model, one picks the population model that generates data with the largest trivial misfit of the analysis model (quantified through the fit index of interest). When selecting an *H*_*1*_ population model, one picks the population model that generates data with the smallest severe misfit of the analysis model. Thus, *H*_*0*_ / *H*_*1*_ population models can differ for different fit indices but are the same across simulation runs.

Although we only applied the fixed method in our examples (again, to keep the exposition simple and help readers understand the basic principles and mechanisms of our simulation-cum-ROC approach), we encourage researchers to consider the random and maximal/minimal methods in future applications of the simulation-cum-ROC approach. We plan to implement these features in later versions. Further, a tutorial on the simulation-cum-ROC approach, including exemplary R code containing the random and maximal/minimal methods, will surely aid the application.

### Guidelines on forms and degrees of misspecification

In the previous section, we have outlined how to incorporate several forms of misspecification (ideally at the same degree of misspecification) into derivations of tailored cutoffs. Both different forms of the same degree and different degrees of the same form of misspecification influence the fit index performance and, thus, tailored cutoffs, as shown by several studies (e.g., Groskurth et al., 2024; McNeish & Wolf, 2023a; Moshagen & Erdfelder, 2016). Thus, the form and degree of misspecification are both relevant for deriving tailored cutoffs, so we want to guide researchers in defining the form and quantifying the degree of misspecification.

Similar to several other authors (e.g., Curran et al., 1996; Hu & Benter, 1998, 1999; McNeish & Wolf, 2023a; Millsap, 2013; Yuan & Bentler, 1997), the analysis models in our examples were (either trivially or severely) misspecified relative to the population models, as they either propose a different model structure or omit specific parameters of a particular size. Thereby, we have already shown different forms of misspecification: A single factor of an analysis model can be misspecified by splitting it into two factors in the population model (e.g., Rosenberg Self-Esteem Scale example), an analysis model of at least two factors can be misspecified by adding cross-loadings^13^ to the population model (e.g., robustness check in Additional File 2 of the Supplementary Online Material), and an analysis model can be misspecified by adding residual covariances to the population model (e.g., Social Desirability-Gamma Short Scale example).

To make the incorporated misspecification (independent of its form) more comparable and thus objective across different scenarios, we can quantify the degree of misspecification in an effect size logic (see also Moshagen & Auerswald, 2018).^14^ For instance, we can quantify the degree of misspecification in terms of the non-centrality parameter (see Jak et al., 2021, who developed a Shiny app for this). The non-centrality parameter can then be transformed into a comparable effect size metric such as ${\chi}^{2}$/*df* or RMSEA, both considered on the population level (Moshagen & Erdfelder, 2016). This effect size helps to quantify and compare degrees of misspecification within or across scenarios. We evaluated the degrees of misspecification induced in our examples in Additional File 3 of the Supplementary Online Material.

However, what constitutes a reasonable population model and a trivial, medium, or severe misspecification of the analysis model relative to that population model depends on many characteristics, such as the research question, study design, and empirical data. Researchers need to justify their definitions of population models based on those characteristics. By requiring that the population models be made explicit, editors, reviewers, and readers of the article can judge the appropriateness of the assumptions about the population model—we believe that this transparency is a major advantage of our simulation-cum-ROC approach.

### Checklist

Overall, the simulation-cum-ROC approach applies to a broad range of empirical settings in which cutoffs must be tailored to the needs of the setting at hand. This also comes with a certain level of subjectivity; the researcher needs to make several decisions, for instance, on the definition of *H*_*0*_ and *H*_*1*_ population models. To guide researchers through this process, we have defined a checklist for evaluating an analysis model with tailored cutoffs using the simulation-cum-ROC approach. This checklist is based on the decisions and pathways outlined throughout this article and can be found in Additional File 4 of the Supplementary Online Material.

## Conclusion

Tailored cutoffs are ideally suited to the empirical setting at hand because they account for the many model, estimation, and data characteristics that can influence fit indices and render fixed cutoffs questionable. This article reviewed four principal approaches researchers can employ to generate tailored cutoffs. We then presented a novel approach, the simulation-cum-ROC approach, that extends previous tailored cutoff approaches, more specifically the dynamic simulation ones, by introducing ROC analysis. Introducing ROC analysis to model fit evaluation is a contribution that uniquely characterizes our approach. It allows for evaluating the performance of fit indices in a given scenario, thus enabling researchers to make informed choices regarding the fit indices on which to rely (or to which to assign the greatest weight). Our approach then derives the most accurate cutoffs for the setting of interest. To the best of our knowledge, the proposed procedure is the only one that allows basing cutoff decisions on balanced type I and type II error rates combined with a performance index for fit indices. The simulation-cum-ROC approach can derive tailored cutoffs for any fit index that a researcher may want to use, including yet-to-be-developed ones. Our procedure to obtain tailored cutoffs comprises three steps (plus fitting and testing the empirical analysis model). We provide a Shiny app and R code to enable researchers to easily generate tailored cutoffs for their empirical problems. We hope to encourage applied researchers to abandon the traditional fixed cutoffs in favor of tailored ones. This will allow them to make valid judgments about model fit and ultimately increase the replicability of research findings. By reviewing possible extensions of our approach, we hope to encourage methodologists to advance further—and help disseminate—the current approaches to generating tailored cutoffs (including our simulation-cum-ROC approach).

## Data Availability

We did not preregister the design and analysis of this article. The data (Nießen et al., 2018, 2020) to reproduce the analysis and results of this article is publicly available on the GESIS SowiDataNet | datorium repository (10.7802/2080 and 10.7802/1752). We also programmed a Shiny app, which is available at https://kg11.shinyapps.io/tailoredcutoffs/. All Additional Files from the Supplementary Online Material of this article are available on the Open Science Framework (https://osf.io/vk94q/).

## Footnotes

1 If not otherwise noted, we refer to the normal-distribution ${\chi}^{2}$ test statistic here, also referred to as *T*_*ML*_ in other articles (e.g., Yuan, 2005).

2 Another way to test whether empirical evidence favors *H*_*0*_ is to look at confidence intervals for fit indices. If those confidence intervals include (or are very close to) 0 indicating perfect fit (for RMSEA and SRMR, alternatively 1 for CFI), empirical evidence favors *H*_*0*_ (e.g., Schermelleh-Engel et al., 2003; or at least one is not able to find evidence against it, Yuan et al., 2016). Confidence intervals have been suggested for several widely used fit indices such as CFI (Cheng & Wu, 2017; Lai, 2019; Yuan et al., 2016; Zhang & Savalei, 2016), RMSEA (Brosseau-Liard et al., 2012; Browne & Cudeck, 1992; Cheng & Wu, 2017; MacCallum et al., 1996; Zhang & Savalei, 2016), and SRMR (Cheng & Wu, 2017; Maydeu-Olivares, 2017; Maydeu-Olivares et al., 2018).

3 One could also add the table-based approach for generating tailored cutoffs to this list (e.g., Groskurth et al., 2024). Reminiscent of looking up critical values for *z*-scores, users read out scenario-specific cutoffs from large tables originating from a prior simulation study. However, as this approach is still very inflexible (as it only allows to read out cutoffs for those scenarios covered in the initial simulation study), we dismissed the approach in our review.

4 MLR (Muthén & Muthén, 1998-2017; Yuan & Bentler, 2000a) is a variant of the most commonly used ML estimator (Bollen, 1989; Jackson et al., 2009). MLR assumes continuous data but corrects the ${\chi}^{2}$ test statistic and standard errors of ML-estimated parameters for non-normality with the help of scaling factors. It yields relatively unbiased model parameters for ordered categorical data with a sufficient number of response options (i.e., five or more; Beauducel & Herzberg, 2006; Rhemtulla et al., 2012). Thus, MLR is very well-known and appropriate in a wide variety of empirical settings, which is why we chose it throughout this article.

5 Readers should not confuse *H*_*1*_ population models, to which we refer here, with saturated *H*_*1*_ models (e.g., Savalei & Rosseel, 2022). Whereas a saturated model perfectly reproduces the structure of the empirical data, the *H*_*1*_ population model used in the simulation-cum-ROC approach is just any reasonable population model compared to which the analysis model of interest is considered misspecified.

6 The simulation-cum-ROC requires two different population models representing hypothetical scenarios on how the data might have come about, encoded in *H*_*0*_ and *H*_*1*_ (following the Neyman–Pearson approach). But these population models are not being compared in the same way that researchers would compare competing analysis models in their empirical data; instead, these populations models are just a “crutch” needed for generating cutoffs. These cutoffs are, in turn, used to make a decision between *H*_*0*_ and *H*_*1*_ for the analysis model tested with empirical data.

7 The AUC might be unexpectedly low even if the models under *H*_*0*_ and *H*_*1*_ actually differ substantially. This can be the case if the empirical, and accordingly the simulated data which is orientated upon the empirical one, has heavy tails. If models are fit to data with heavy tails, parameter estimates can be inefficient, which might lead to a bad discrimination ability of fit indices between the analysis model under *H*_*0*_ and *H*_*1*_. Robust methods that downweigh cases in the tail area, applied to the empirical and simulated data, lead to more efficient parameter estimates. Thus, those methods can help to improve the separation of fit index values under *H*_*0*_ and *H*_*1*_ (see Yuan et al., 2004, on identifying the optimal robust procedure for the data of interest).

8 The simulation-cum-ROC approach also allows to consider one error as more problematic than the other by changing the *metric* for cutpointr (Thiele & Hirschfeld, 2021) in the R code to either *sens_constrain* (if the type II error is considered more problematic) or *spec_constrain* (if the type I error is considered more problematic).

9 Exceptions may occur where the fit index with the highest AUC does not have the cutoff with the highest accuracy. For instance, the fit index with the highest AUC does not need to have the cutoff with the highest accuracy if AUCs of different fit indices are only marginally different from each other.

10 Olvera Astivia and Zumbo (2015) scrutinized Vale and Maurelli’s method and found that estimates of skewness and kurtosis were downward-biased, especially in small samples. We still relied on Vale and Maurelli’s method to obtain multivariate non-normal data as it is the standard method in the simsem package (Pornprasertmanit et al., 2021) used here. However, researchers can adapt the code provided in this article estimating multivariate non-normal data using other functions (e.g., the functions of the R package covsim, Grønneberg et al., 2022, can be called in simsem’s function to simulate data, *sim*, but covsim’s functions require to specify a population variance-covariance matrix instead of a population model).

11 As evident from Fig. 6, we take the mean of the optimal cutoff and the next highest fit index value as a revised optimal cutoff (or the next lowest fit index value when lower values imply worse fit, such as for CFI; Thiele & Hirschfeld, 2021). To find an optimal cutoff, the algorithm first uses each fit index value as a potential cutoff starting with those indicating good (e.g., CFI = 1.00, RMSEA = 0.00) to poor model fit (e.g., CFI = 0.00, RMSEA = 1.00). It evaluates sensitivity and specificity at each potential cutoff. Then it selects the fit index value with the highest sum of sensitivity and specificity as an optimal cutoff. For non-overlapping distributions, both the worst fit index value from correctly specified models and the best fit index value from misspecified models have the highest sum of sensitivity and specificity. The algorithm would, thus, choose the worst fit index value from correctly specified models as an optimal cutoff. It is the first value with the highest sum of sensitivity and specificity (because the algorithm starts from good to poor model fit). We let the algorithm take the mean between the optimal cutpoint (i.e., the worst fit index value from correctly specified models in non-overlapping distributions) and the next value (i.e., the best fit index value from misspecified models in non-overlapping distributions) as a revised optimal cutoff to avoid bias in favor of correctly specified models. By this, we obtain a revised optimal cutpoint, which is the mean of the optimal cutpoint and the next fit index value.

12 As one reviewer correctly pointed out, RMSEA is just a transformation of ${\chi}^{2}$ (e.g., Moshagen & Erdfelder, 2016). RMSEA can therefore be considered redundant because its performance in terms of the AUC will be the same as that of the ${\chi}^{2}$. Nonetheless, we decided to generate cutoffs for both the ${\chi}^{2}$ and the RMSEA in the examples because both are regularly used for model evaluation (Jackson et al., 2009).

13 Whereas misspecification increases with a larger magnitude of unmodeled cross-loadings, adding more unmodeled cross-loadings to the population model leads to “lower” misspecification in the analysis model (if factors are correlated). Other parameters can compensate for the added cross-loadings (Groskurth et al., 2024).

14 Some authors (Cudeck & Browne, 1992; Yuan et al., 2007) have proposed to directly define misspecification in terms of an effect size logic, without defining the particular form (or location) of misspecification. They define (or approximate) the population variance-covariance matrix at a given distance from the analysis model structure.

## Code availability

The code to reproduce this article’s simulation, analysis, and results is available in Additional File 1 of the Supplementary Online Material.

## Declarations

### Prior dissemination

We have published the article as a preprint on PsyArXiv (10.31234/osf.io/62j89) and advertised it on ResearchGate and Twitter. We have also published a previous version of this article on MADOC (https://madoc.bib.uni-mannheim.de/64707/), an online repository of the University of Mannheim, as part of Katharina Groskurth’s doctoral thesis. Further, we narratively presented prior versions of this article at three online conferences in 2021: (1) Meeting of the Working Group on Structural Equation Modelling (SEM) on March 18–19, 2021, (2) International Meeting of the Psychometric Society (IMPS) on July 19–23, 2021, and (3) the 15th Conference of the Section ‘Methods and Evaluation’ in the German Psychological Society (FGME) on September 15–17, 2021. We confirm that this article has not been externally presented, submitted, or published elsewhere.
